# Critical Assessment of Intrinsic Antibacterial Properties and Photothermal Therapy Potential of MXene Nanosheets

**DOI:** 10.1021/acsanm.5c04961

**Published:** 2026-01-13

**Authors:** Viktoriia Korniienko, Oleksiy Gogotsi, Yuliia Varava, Baiba Zandersone, Volodymyr Deineka, Yevheniia Husak, Kateryna Diedkova, Oleksandr Solodovnyk, Vjacheslav Kukurika, Serhii Dukhnovskiy, Roman Moskalenko, Ivan Baginskiy, Oksana Petrichenko, Oksana Sulaieva, Olena Haidamak, Pavlo Shubin, Veronika Zahorodna, Błażej Anastaziak, Emerson Coy, Igor Iatsunskyi, Yury Gogotsi, Maksym Pogorielov

**Affiliations:** † Institute of Atomic Physics and Spectroscopy, Faculty of Science and Technology, 61769 University of Latvia, Jelgavas 3, LV-1004 Riga, Latvia; ‡ Biomedical Research Centre, 187506 Sumy State University, 40007 Sumy, Ukraine; § Materials Research Centre, 3 Krzhizhanovskogo Str., 03142 Kyiv, Ukraine; ∥ NanoCarbonTech, Rubiez 46, 61-612 Poznan, Poland; ⊥ Faculty of Chemistry, Silesian University of Technology, Strzody 9, 44-100 Gliwice, Poland; # Werba Medical, 40035 Sumy, Ukraine; ¶ Department of Physics, Faculty of Science and Technology, University of Latvia, Jelgavas 3, LV-1004 Riga, Latvia; ∇ Medical Laboratory CSD, Vasylkivska 45, 02000 Kyiv, Ukraine; ○ NanoBioMedical Centre, 49562 Adam Mickiewicz University, 3, Wszechnicy Piastowskiej Str., 61-614 Poznan, Poland; ⧫ A. J. Drexel Nanomaterials Institute, and Department of Materials Science and Engineering, Drexel University, Philadelphia, Pennsylvania 19104, United States

**Keywords:** MXene, antibacterial, biocompatibility, ROS-mediated damage, photothermal therapy, targeted delivery

## Abstract

MXenes are well-known as highly biocompatible two-dimensional nanomaterials with a wide range of biomedical applications, including antibacterial strategies. However, the coexistence of high biocompatibility and reported strong antibacterial effects presents a fundamental contradiction that requires critical evaluation. In this study, we systematically investigated the antibacterial properties of pure Ti_3_C_2_T_*x*_, Nb_2_CT_*x*_, V_2_CT_*x*_, and Ti_3_CNT_*x*_ MXene nanosheets of varying flake sizes using multiple in vitro assays and an in vivo wound model. High-resolution structural and chemical characterizations confirmed the use of high-quality, minimally oxidized MXene samples with well-defined surface terminations. Despite using multiple evaluation methods, including disk diffusion, broth microdilution, time-kill kinetics, ROS quantification, and electron microscopy, no significant antibacterial effects were observed at subtoxic concentrations. Furthermore, neither reactive oxygen species-mediated damage nor the hypothesized “nano-knife” mechanical disruption mechanism could be confirmed. This suggests that the previous observations of antibacterial properties resulted from incomplete removal of etching products or partial oxidation of MXene nanosheets. In contrast, we demonstrate that MXene-assisted photothermal therapy (PTT) under near-infrared laser irradiation offers highly effective and selective bacterial ablation. Ti_3_C_2_T_*x*_ MXene exhibited strong photothermal performance, achieving complete bacterial killing in vitro and significant wound healing efficacy in an in vivo rat model. Targeted PTT using antibody-functionalized MXene nanosheets enabled the eradication of *Escherichia coli* while sparing nontarget bacteria. These findings suggest that while intrinsic antibacterial properties of pristine MXenes are limited, their biocompatibility and photothermal responsiveness make them promising platforms for next-generation, externally triggered antibacterial therapies.

## Graphical abstract

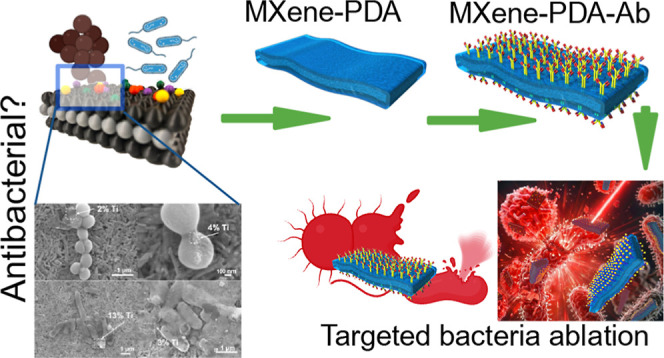

## 1 Introduction

MXenes, a large family of two-dimensional (2D) materials composed of transition metal carbides, nitrides, or carbonitrides, have garnered significant attention for their unique properties, including high electrical conductivity, large surface area, and controlled surface chemistry.^1^ In particular, their antibacterial properties have become an area of increasing interest, yet despite several studies suggesting antimicrobial activity, the extent of their antibacterial properties remains uncertain, still being under investigation. Since the first report in 2016 demonstrating the antibacterial activity of MXene nanosheets,^2^ numerous studies have confirmed their broad-spectrum antibacterial potential.^3–5^ The first report on their antibacterial activity, published in 2016, suggested that single- and few-layer Ti_3_C_2_T_*x*_ nanosheets exhibited strong antibacterial effects against *Escherichia coli* and *Bacillus subtilis*. The antibacterial activity was linked to the delamination of MXenes, which increased their surface area and created sharp edges, enhancing their ability to interact with bacterial cells. Nanosheets with sharp edges were found to penetrate the bacterial cell membrane more effectively, causing damage to the cytoplasmic components, including DNA.^2^

Recent studies have suggested that the antibacterial activity of MXenes is influenced by multiple physicochemical parameters, including lateral size, contact angle, sheet thickness, and surface functionalization. Notably, delaminated Ti_3_C_2_T_*x*_ nanosheets with smaller lateral dimensions have been shown to enter microbial cells either via direct physical penetration or through endocytosis-like mechanisms, analogous to those described for graphene oxide nanosheets.^6^ Furthermore, MXene flakes with reduced size and increased defect density have been reported to induce oxidative stress, thereby enhancing their bactericidal efficacy.^7^ In another study, the antibacterial mechanisms of ultrasonicated Ti_3_C_2_T_*x*_ MXene nanosheets were investigated, highlighting how variations in lateral size and edge sharpness induced by controlled ultrasonication enhanced their antimicrobial efficacy.^8^ Also, the antibacterial activity of MXenes was primarily attributed to their surface reactivity, which facilitates interactions with bacterial cell membranes, potentially disrupting cell integrity and leading to bacterial death. MXenes obtained by selective etching in fluoride-containing acidic solutions possess functional groups such as hydroxyl (−OH), oxygen (−O), and fluorine (−F) on their surfaces, enhancing their hydrophilicity and increasing the likelihood of interactions with bacterial cells.^9^ The antibacterial properties of MXenes were reported to be enhanced by various factors, including their surface functionalization and structural modifications. Freshly synthesized MXenes carry abundant hydrophilic surface terminations (T_*x*_ = −OH, −O, −F, etc.), which improve their water dispersibility and contact with bacterial cells. These inherent surface functional groups not only enhance the material’s activity but also provide versatile sites for further modification. The aging process of Ti_3_C_2_T_*x*_ membranes has been shown to improve their antibacterial activity due to the formation of TiO_2_ nanocrystals on the surface,^7^ a phenomenon also observed in titanium substrates coated with TiO_2_.^10^ This effect is believed to arise from the synergistic interaction between Ti_3_C_2_T_*x*_ nanosheets and TiO_2_/C structures formed during surface oxidation, leading to reduced bacterial adhesion and increased antimicrobial efficacy.^7^ Oxidation of Ti_3_C_2_T_*x*_ MXene results in reduced conductivity, spectral changes, and the emergence of titanium oxides. Experimental studies have shown that Ti_3_C_2_T_*x*_ undergoes gradual degradation in air, aqueous dispersions, and solid films, with the fastest oxidation occurring in liquid media and being further accelerated by UV exposure.^11^ Long-term monitoring demonstrates progressive formation of mixed TiO_*x*_ phases during aging and significant changes in electrochemical behavior.^12^ Additionally, the stability of MXene films in environments relevant to bioelectronics has been shown to depend strongly on humidity, storage conditions, and film-processing methods.^13^ These data underscore the importance of rigorous control over MXene oxidation to ensure consistent antibacterial performance and biocompatibility.

Researchers have leveraged MXene nanosheet surface functionalities to tune antibacterial outcomes by attaching polymers, metal nanoparticles, or other antimicrobial moieties.^14–17^ Such surface modifications can increase the stability of MXene colloids, target bacteria more effectively, or add new bactericidal functions. For instance, exploiting MXenes’ surface groups to load silver nanoparticles or antibiotics has yielded composites with superior antibacterial efficacy compared to MXene alone.^4^ The antibacterial effect of MXenes was also reported to be time-dependent, with longer incubation times enhancing their bactericidal activity. The proposed mechanism involved the sharp edges of the nanosheets interacting directly with the bacterial cell wall, leading to mechanical disruption. This mechanism may be particularly effective for Gram-positive bacteria, where the cell wall is thinner and more vulnerable to mechanical disruption. For example, extending the contact time to 8 h with 90 nm MXenes improved antibacterial activity against both *E. coli* and *B. subtilis* to over 95%.^17^

Several experimentally supported mechanisms can explain the antibacterial activity of MXene nanosheets. First, the sharp, atomically thin edges of MXene nanosheets can physically pierce and sever bacterial cell walls (commonly referred to as the “nano-knife” effect), resulting in membrane rupture, cytoplasmic leakage, and ultimately cell lysis.^4^ Second, MXenes can induce oxidative stress in bacterial cells by generating reactive oxygen species (ROS), such as singlet oxygen, superoxide, and peroxides. This oxidative stress damages bacterial membranes, proteins, and DNA, significantly reducing bacterial viability. Notably, delaminated Nb_4_C_3_T_*x*_ MXene with the lateral sheet size 183 nm has been shown to induce a higher level of oxidative stress compared to the 160 nm-size one in ROS-independent assays, correlating with its slightly enhanced antibacterial activity.^18^ Third, MXenes exhibit strong photothermal conversion capabilities; upon exposure to near-infrared (NIR) irradiation, they generate localized hyperthermia that effectively kills bacteria and can synergistically amplify other antibacterial effects. Rosenkranz et al. (2021) demonstrated that few-layered Ti_3_C_2_T_*x*_ nanosheets exhibit significantly stronger antibacterial effects against *E. coli* and *Staphylococcus aureus* under laser irradiation compared to multilayered Ti_3_C_2_T_*x*_, due to their greater disruption of bacterial membranes and higher leakage of intracellular contents.^19^ Furthermore, the large surface area and tunable surface chemistry of MXenes enable strong interactions with bacterial membranes. MXene-based composites can also be functionalized to release antibacterial metal ions. For example, the incorporation of gold nanoparticles into MXene structures has been shown to enhance antibacterial efficacy against both Gram-positive and Gram-negative bacteria.^20^ Collectively, these mechanisms, including physical membrane disruption, ROS-mediated oxidative damage, photothermal ablation, and metal ion release, underscore the notable and multifaceted antibacterial properties of MXenes.

At the same time, several studies have reported a lack of antibacterial activity in certain MXene formulations. Jastrzębska et al. demonstrated that Ti_2_CT_*x*_ exhibits no biocidal activity against various Gram-positive bacteria, including *B. subtilis*, *S. aureus*, and *Sarcina* species, underscoring the critical role of atomic structure and stoichiometry (e.g., Ti_2_CT_*x*_ vs Ti_3_C_2_T_*x*_) in determining the antimicrobial efficacy of MXenes.^21^ Similarly, Warsi et al. (2022) reported that Ti_3_C_2_T_*x*_ alone did not exhibit significant antibacterial activity; only after adding tungsten trioxide (WO_3_) the composite material demonstrate antibacterial effects against both Gram-positive and Gram-negative bacteria.^14^ Furthermore, Cheng et al. (2023) showed that MXene-based membranes required functionalization with other materials, such as graphene oxide (GO), oxygen-doped graphitic carbon nitride (O-*g*-C_3_N_4_), or bismuth oxychloride (BiOCl), to exhibit enhanced antibacterial properties, highlighting the insufficient biocidal activity of pure MXene.^22^

Despite the documented antibacterial effects, MXenes are often reported to remain biocompatible at similarly effective concentrations. Many studies have shown that MXenes, regardless of their chemical composition, are nontoxic to mammalian cells at concentrations up to 100 μg/mL,^23–25^ with some reports indicating biocompatibility of Ti_3_C_2_T_*x*_ at concentrations as high as 250–500 μg/mL.^26^ These findings conflict with data on their antibacterial efficacy and warrant further clarification. Notably, several studies have demonstrated that mammalian cells, including cancer cells, can be more sensitive to nanomaterials than bacterial cells, which complicates the interpretation of cytotoxicity data.^27,28^ Mechanisms underlying the antibacterial effects of MXenes, such as physical membrane disruption (“nano-knife” effect) and ROS-mediated damage, may also contribute to cytotoxicity in mammalian systems, thereby limiting their safe application as antibacterial agents. Furthermore, existing studies often overlook critical variables that influence both toxicity and antibacterial potential, including protein corona formation, chemical impurities, and the oxidation state of MXenes. These aspects must be carefully considered to accurately assess the biomedical utility of MXenes.

In this study, we present a comprehensive investigation of the antibacterial properties of several typical MXene nanosheets (Ti_3_C_2_T_*x*_, Ti_3_CNT_*x*_, Nb_2_CT_*x*_, and V_2_CT_*x*_) using both classical and modified bacteriological assays and correlate these activities with their structural and chemical features. We explore key hypotheses regarding MXene antibacterial mechanisms and evaluate how material properties such as lateral size, oxidation state, and surface terminations affect both biocompatibility and antibacterial activity. We focused on pure high-quality MXenes to understand if the bactericidal effect was intrinsic or caused by external factors. Finally, we propose a strategy for the safe and effective use of MXenes in photothermal antibacterial applications, including targeted delivery via antibody-functionalized complexes.

## 2 Experimental Section

### 2.1 MXene Synthesis

Titanium-based MXenes Ti_3_C_2_T_*x*_ and Ti_3_CNT_*x*_ (*T* represents terminations, such as, –OH, = O or –F) were prepared by selective wet chemical etching of aluminum (Al) layer from Ti_3_AlC_2_ and Ti_3_AlCN ternary carbides and carbonitrides (MAX-phases), respectively, using diluted hydrofluoric acid (HF) as an etching agent either in free form or formed in situ via reaction of lithium fluoride (LiF) with hydrochloric acid HCl, known as MILD-method.^9^ V_2_CT_*x*_ and Nb_2_CT_*x*_ MXenes were prepared by a similar wet etching technique using V_2_AlC and Nb_2_AlC MAX-phases by a HF/HCl mixture, but with a relatively higher concentration of HF.^29^ All employed MAX-phases, Ti_3_AlC_2_, Ti_3_AlCN, V_2_AlC, and Nb_2_AlC, had particle size below 40 μm and prior to etching were washed with 14% hydrochloric acid (HCl) at 50 °C for 6 h in order to clean MAX-phases from intermetallic compounds.

#### 2.1.1 Synthesis of Ti_3_C_2_T_*x*_ and Ti_3_CNT_*x*_ via Low-Fluoride (<5%) MILD Method

The etching solution was prepared by mixing 200 mL of 0.9 M HCl and 16 g of LiF in a polypropylene vessel with a volume of 500 mL. The vessel was put in oil bath heated to 35 °C on magnetic stirrer. 2.5 cm × 1 cm magnetic rod was used for stirring. Ten g of Ti_3_AlC_2_ or Ti_3_AlCN MAX-phase were slowly added to the etching solution under stirring. The mixture was kept at 35 °C under constant stirring for 24 h.

#### 2.1.2 Synthesis of V_2_CT_*x*_ and Nb_2_CT_*x*_ MXenes

The etching solutions were prepared by mixing 36% HCl and 50% HF in a 4:6 ratio in Teflon vessels with a volume of 250 mL. The vessel was put in an oil bath heated to 50 °C on a magnetic stirrer. A 2.5 cm × 1 cm magnetic rod was used for stirring. Four g of V_2_AlC or Nb_2_AlC MAX-phase were slowly added to 80 g of etching solution. The mixture was kept at 50 °C under constant stirring for 72 h.

#### 2.1.3 Rinsing after Etching

The obtained multilayer Ti_3_C_2_T_*x*_, Ti_3_CNT_*x*_, V_2_CT_*x*,_ and Nb_2_CT_*x*_ MXenes were cleaned from excess acids via repetitive cycles of MXene sedimentation via centrifugation at 2800 rcf for 10 min. Acid- and salt-containing supernatant was discarded, and the multilayer MXene sediment was dispersed again in a fresh portion of DI water, then centrifuged again. The procedure was repeated until the pH value of the supernatant reached ∼6.

#### 2.1.4 Delamination

To delaminate the etched MXenes to separate 2D sheets dispersible in water, the following delamination procedures were employed. Delamination of Ti_3_C_2_T_*x*_ and Ti_3_CNT_*x*_ was performed via intercalation of lithium cations. The etched multilayer Ti_3_C_2_T_*x*_ or Ti_3_CNT_*x*_ MXene was added to a solution of LiCl with a concentration of 50 g per liter, considering a ratio of 1 g of initial Ti_3_AlC_2_ or Ti_3_AlCN MAX-phase per 20 mL of LiCl solution. The mixture was stirred for 24 h at 35 °C. Delamination of V_2_CT_*x*_ and Nb_2_CT_*x*_ MXenes was achieved via intercalation of an organic base, tetrabutylammonium hydroxide (TBAOH). The etched multilayer V_2_CT_*x*_ and Nb_2_CT_*x*_ MXenes were added to an aqueous solution of TBAOH (5 wt %), considering a ratio of 1 g of initial V_2_AlC or Nb_2_AlC MAX-phase per 20 mL of solution.

#### 2.1.5 MXene Extraction and Separation According to Size

After delamination, MXenes were rinsed with water to remove excess intercalant and transfer single-layer MXenes into a colloidal solution in water through repetitive cycles of sedimentation of multilayer MXenes, via centrifuging at 2000 rcf (Eppendorf 5702) for 10 min, followed by separating the MXene-containing supernatant and dispersion of sediment in a fresh portion of DI-water. As the supernatant after centrifuging maintained a dense black color, delaminated single-layer MXene was transferred into colloidal form, and it was collected and stored.

MXenes were sedimented from the colloidal solution via centrifugation at 2500 rcf (Eppendorf 5702) for 20 min to form a concentrated MXene slurry with large-size flakes (*L*). The remaining supernatant containing lighter (smaller flake size) MXenes was also collected. Accumulated MXene sediments were rinsed with DI water three times via repetitive dispersing and centrifugation under conditions similar to initial sedimentation to clean MXene from possible impurities. The accumulated supernatant with lighter MXenes was first centrifuged at 4200 rcf (Janetzki T23) to sediment and separate medium-sized flakes. The remaining supernatant was collected again and centrifuged at 6500 rcf (Janetzki T23) to sediment small-sized MXene flakes (*S*). Ti_3_C_2_T_*x*_ MXenes were stored in the form of a concentrated water-based slurry, while more prone to oxidation Ti_3_CNT_*x*_, V_2_CT_*x*_, and Nb_2_CT_*x*_ MXenes were dispersed in 2-propanol, sedimented, and stored under 2-propanol.

### 2.2 Characterization

A detailed analysis of the structural and morphological features of the synthesized MXene materials was conducted using atomic force microscopy (AFM), scanning electron microscopy (SEM) combined with energy-dispersive X-ray spectroscopy (EDX), transmission electron microscopy (TEM) with elemental mapping, and Raman spectroscopy. AFM imaging was carried out with a Bruker Dimension Icon system in tapping mode to assess surface topography and flake thickness. SEM observations and compositional analysis were performed using a JEOL JSM-7001F microscope operated at accelerating voltages of 5 kV. For high-resolution imaging, transmission electron microscopy (TEM) was conducted using a JEOL 2100F instrument at an acceleration voltage of 200 kV. Specimens were prepared by depositing diluted MXene suspensions onto copper grids coated with lacey carbon. Raman measurements were performed using a Renishaw inVia confocal Raman system equipped with a 785 nm excitation laser. A 50× objective lens was used to focus the beam on the sample surface, and the system provided a spectral resolution of approximately 1 cm^–1^.

### 2.3 Cytotoxicity Assessment of MXenes on Human Cell Lines

The cytotoxic effects of various types of MXenes were evaluated using a human keratinocyte cell line (HaCaT) and a human melanoma cell line (MaMel 8b), both obtained from the cell collection at the University of Latvia. Cells were cultured in Dulbecco’s Modified Eagle’s Medium (DMEM) supplemented with 10% fetal bovine serum (FBS), 2.0 mM l-glutamine (all from Sigma-Aldrich, Inc.), 100 U/mL penicillin, 100 μg/mL streptomycin, and 2.5 μg/mL amphotericin B (Gibco, USA). Cultures were maintained at 37 °C in a humidified atmosphere containing 5% CO_2_.

Once the cells reached approximately 85% confluence in 75 cm^2^ flasks, they were trypsinized and seeded into 96-well plates at densities of 7000 cells/cm^2^ for HaCaT and 9000 cells/cm^2^ for MaMel 8b. After 4 and 24 h of incubation, the culture medium was removed and replaced with fresh complete medium containing different concentrations of MXenes (6.25, 12.5, 25, 50, and 100 μg/mL). Each concentration was tested in triplicate. Control wells without MXenes were included for comparison and to calculate the percentage of cell viability. Cells were coincubated with MXenes for 24 h. Following incubation, wells were gently washed with phosphate-buffered saline (PBS) to remove unbound nanoparticles and cellular debris. Subsequently, 200 μL of fresh complete medium was added to each well.

Cell viability and proliferation were assessed using the Resazurin reduction assay on days 1, 3, and 6. At each time point, the medium was removed, wells were rinsed with PBS, and fresh medium containing Resazurin at a final concentration of 15 μg/mL was added. Plates were incubated for 2 h in a CO_2_ incubator. A cell-free medium containing Resazurin was used as a negative control to account for background fluorescence. After incubation, 100 μL aliquots of the Resazurin-containing medium were transferred to a new black 96-well plate. Fluorescence was measured using a TECAN Infinite M200 Pro microplate reader (Switzerland) at 560 nm excitation and 590 nm emission wavelengths.

### 2.4 Diffusion-Based Assays

Diffusion-based assays, as well as all other in vitro bacteriological experiments, were performed using *S. aureus* (ATCC 29213, Manassas, VA, USA) and *E. coli* (ATCC 25922, Manassas, VA, USA) strains.

#### 2.4.1 Agar Disk Diffusion Method

Bacterial cultures were grown overnight, diluted to an appropriate concentration (10^8^ CFU/mL) in Mueller–Hinton broth (MHB, SKU 70192, Sigma-Aldrich, St. Louis, MO, USA) and spread uniformly onto Mueller–Hinton agar plates (MHA, SKU 70191, Sigma-Aldrich, St. Louis, MO, USA). In this assay, bacterial cultures are grown on nutrient agar plates, and filter paper disks (about 6 mm in diameter) are impregnated with varying concentrations of MXenes (20 μL per disk). Typically, concentrations range from 125 μg/mL to 2000 μg/mL. The disks are then placed onto the inoculated agar surface, ensuring even distribution of bacteria. The plates are incubated at 37 °C for 24 h. The antibacterial effect is assessed by measuring the diameter of the inhibition zone, the clear area surrounding the disk where bacterial growth is hindered. Ceftazidime CAZ 30 μg Antibiotic Disc was used as a control. A larger inhibition zone indicates stronger antibacterial activity, providing a qualitative measure of the antibacterial potency of MXenes.

#### 2.4.2 Agar Drop Diffusion Method

Drop diffusion assays were performed by incorporating MXenes into agar plates. The microorganisms tested were spread over an MH agar plate surface. Tests were carried out by application of a 3 μL drop of the sample placed on the surface of the plate. The antimicrobial activity of MXene against the bacteria was indicated by the inhibition zone diameter (cm) around the point where each sample drop was placed on the inoculated medium surface. Three μL of the antibiotic ciprinol (10 mg/mL solution) was used as a positive control. Plates were incubated at 37 °C for 24 h. The diameter of the inhibition zone (cm) was measured after the end of the incubation period.

### 2.5 Dilution Methods

#### 2.5.1 Minimum Inhibitory Concentration (MIC) and Minimum Bactericidal Concentration (MBC) Determination

The MIC was determined using a serial dilution method in a microtiter plate format. Bacterial suspensions were exposed to varying concentrations of MXenes, ranging from 125 μg/mL to 2000 μg/mL. The plates were incubated at 37 ± 1 °C for 24 h, and bacterial growth was visually assessed using transmitted light to observe the presence or absence of turbidity. The lowest concentration at which no visible growth was observed was designated as the MIC, expressed in μg/mL. To ensure reproducibility, some tests were carried out in triplicate.

Additionally, the Resazurin microtiter MIC assay was conducted. To prevent self-reduction of resazurin by MXene, 100 μL of the bacterial suspension was transferred to sterile 96-well plates. Sterile broth was used as the negative control, while bacterial broth served as the positive control. A 10% v/v concentration of commercially available resazurin solution (Sigma-Aldrich, USA) was added. The viability of the bacteria was assessed based on the color change of resazurin from blue/purple to fluorescent pink. The bacteria were incubated at 37 °C for 2 h, until the color change was visible. The lowest concentration of MXenes with no color change in resazurin was defined as the MIC. Fluorimetry was performed using the Tecan Infinite M Nano Plus Multi Detection Microplate Reader (Tecan Trading AG, Switzerland), with excitation at 544 nm and emission at 590 nm to measure resazurin metabolization by the bacteria. Three replicates were performed for each sample concentration.

For MBC determination, aliquots from the serial dilution suspension corresponding to the MIC and the two or three preceding dilutions were transferred to plates of solid growth medium, which were then incubated for 24 h at 37 °C. After incubation, the plates were examined for microbial growth. The dilution at which no growth was observed was recorded as the MBC.

#### 2.5.2 Concentration-Dependent Time-Kill Tests

The inhibitory effect of Ti_3_C_2_T_*x*_ MXene was evaluated starting at a concentration of 1× MIC (2000 μg/mL). MXene suspensions were added to the top row of a 96-well plate and subjected to a 2-fold serial dilution, resulting in final concentrations ranging from 2000 μg/mL to 125 μg/mL. The experiment was conducted in a broth culture medium using a bacterial suspension of 5 × 10^5^ CFU/mL, with the same bacterial concentration serving as the growth control. Incubation was carried out under appropriate conditions for 4 and 24 h. The percentage of dead cells was determined relative to the growth control by quantifying viable bacterial counts (CFU/mL) using the agar plate count method.

A bactericidal effect was defined as 90% lethality within 6 h, corresponding to 99.9% lethality after 24 h. In our research, we used a 4 h incubation period to compare our results with previous studies, as the 4 h time point is one of the most frequently reported in studies on MXene’s antibacterial properties.

To expand the understanding of the time-dependent antibacterial activity of MXene at concentration of 100 μg/mL, we applied a spectrophotometric growth rate analysis. This method allowed us to monitor bacterial growth dynamics in real-time by measuring absorbance at 600 nm at regular intervals. Microbial cell numbers were adjusted to approximately 10^5^ CFU/mL and added to the wells. Microbial growth was monitored using a spectrophotometer (Tecan Infinite 200 PRO Multimode Microplate Reader) by measuring absorbance at 600 nm every hour, following a 100 rpm orbital shake before each measurement. The assay was conducted under continuous incubation at 37 °C for 24 h. The initial absorbance was used as a blank. The absorbance of nanoparticles at different concentrations was accounted for during data analysis. Each sample concentration was tested in triplicate, and the mean absorbance values were plotted to generate time-kill curves, which were compared to positive and negative controls.

Additionally, we applied the HB&L (High Bacterial Load) analyzer (Alifax, Italy), a sophisticated, light-scattering system designed for rapid bacterial growth detection directly in liquid media. It operates on the principle of laser nephelometry: a laser beam passes through a sample, and as bacteria grow, they increasingly scatter light. The analyzer measures this real-time increase in scattered light intensity to detect and quantify microbial proliferation with high sensitivity and specificity. It functions as a fully automated, closed system. In our tests, 24 h cultures of *S. aureus* and *E. coli*were used, each starting at an initial concentration of 10^6^ CFU/mL, and exposed to MXene at concentrations of 200, 50, and 25 μg/mL.

#### 2.5.3 Continuous Rotation Assay

The experiment was conducted to evaluate the interaction of MXene nanomaterial with bacterial cultures of *S. aureus* and *E. coli*. Bacterial suspensions were prepared in Mueller–Hinton Broth (MHB) at a concentration of 10^6^ CFU/mL. MXene was used at a final concentration of 400 μg/mL. For each test condition, 500 μL of the MXene suspension in MHB was added to a 2 mL Eppendorf tube, followed by 500 μL of the bacterial suspension. The mixtures were incubated at 37 °C for 24 h under continuous rotation to prevent sedimentation of the MXene particles on the tube walls and to ensure homogeneous exposure. After incubation, 30 μL from each tube was plated onto Mueller–Hinton Agar (MHA) plates and further incubated for 24 h at 37 °C. Bacterial growth was visually assessed after incubation. The setup allowed evaluation of the potential antimicrobial activity of MXenes under dynamic conditions (Supporting Information, Figure S1a).

### 2.6 Bacterial Biofilm Viability Assay by Crystal Violet Staining

The biofilm was established by inoculating a culture of *E. coli* or *S. aureus* into the nutrient broth at a concentration of 10^5^ CFU/mL. Subsequently, 100 μL of the bacterial suspension in Mueller–Hinton broth (MHB) medium with an optical density (OD_600_) of 0.5 was transferred to 96-well plates, allowing the bacteria to adhere and form biofilms. This incubation period lasted 24 h to ensure sufficient biofilm maturation. After biofilm formation, Ti_3_C_2_T_*x*_ MXene at a concentration of 2000 μg/mL was applied directly to the biofilm. The treatment was conducted at 37 °C for 24 h, while control wells remained untreated.

Following treatment, planktonic cells were aspirated, and the biofilms were washed three times with 1000 μL of phosphate-buffered saline (PBS). The biofilms were then stained with 200 μL per well of 0.1% (w/v) crystal violet solution for 15 min. Excess crystal violet (CV) was removed, and the plates were washed three times with 100 μL of PBS and air-dried for 30 min. To quantify biofilm biomass, the bound crystal violet was dissolved in 200 μL per well of 80% ethanol. Absorbance was measured at 590 nm (*A*_590_) using a Multiskan FC spectrophotometer (Thermo Fisher Scientific, Waltham, MA, USA).

Reduction in biofilm mass ratio (percentage reduction in biofilm mass by comparing the optical density (OD) of wells treated with MXene to that of untreated wells) was calculated using Formula [Disp-formula eq1]

1 $$Survival,\%=\frac{OD\;of\;treated\;wells}{OD\;of\;untreated\;wells}\times 100\%$$

### 2.7 Reactive Oxygen Species (ROS) Generation Assay

To explore the potential mechanism of MXene cytotoxicity, intracellular reactive oxygen species (ROS) levels were measured using the nonspecific fluorescent probe 2′,7′-dichlorofluorescein diacetate (DCF-DA).^30^ *E. coli* bacterial suspensions were prepared at a 1.0 McFarland standard from overnight culture, and 500 μL of 100 μg/mL MXene solution in Mueller–Hinton Broth (MHB) was added to 500 μL of the bacterial suspension. The mixture was incubated at 37 °C for 5, 15, 30, and 90 min. After incubation, the suspension was centrifuged, and the supernatant was discarded. The pellet was resuspended in 1 mL PBS, centrifuged again, and mixed with 1 mL of DCF-DA reagent solution. The suspension was incubated in the dark at 37 °C for 30 min. After incubation, the supernatant was discarded, and the final pellet was resuspended in 100 μL of PBS. The sample was transferred to a black 96-well plate, and fluorescence intensity was measured using a microplate reader with excitation at 485 nm and emission at 535 nm. This method allows for the measurement of intracellular ROS levels, providing insight into the oxidative stress induced by MXene treatment.

### 2.8 SEM and TEM Analyses of MXene-Bacteria Interaction

SEM analysis was conducted to examine the MXene-bacteria interaction (Supporting Information, Figure S2). A Phenom ProX, (Phenom-World BV, Eindhoven, The Netherlands) and a JEOL JSM-7001F microscopes equipped with an energy-dispersive X-ray spectrometer (EDX) were used for sample observations. TEM investigation was conducted using a JEOL 2100F instrument. Bacterial suspensions were exposed to MXenes at a concentration up to 2000 μg/mL in a microtiter plate and incubated at 37 ± 1 °C for 24 h. Following incubation, the contents of the wells were transferred to Eppendorf tubes and fixed with 1% glutaraldehyde (Sigma-Aldrich) in phosphate-buffered saline (PBS, pH 7.4) for 15 min. Each subsequent step involved centrifugation to remove residual liquids, followed by the addition of fresh solutions and resuspension, ensuring that the bacterial cells remained well-separated. After the initial fixation, samples were centrifuged again, and the supernatant was removed. A second fixation was performed using 1 mL of 1% glutaraldehyde in PBS, followed by an additional 15 min incubation. The cells were then washed three times with PBS (pH 7.4), and dehydration was carried out using a graded ethanol series (25%, 50%, 80%, and 96%). For SEM imaging, a 5 μL drop of each sample suspension was placed on carbon tape, allowed to dry, and then coated with a 5 nm layer of gold by sputtering.

### 2.9 Calculation of MXene-Bacterial Interaction

It was necessary to calculate the amount of Ti_3_C_2_T_*x*_ MXene flakes per each bacterium in volume unit of medium and also distances between MXene flakes in the solution. To simplify the calculations, the shape of flakes was considered to be rectangular. Concentration of Ti_3_C_2_T_*x*_ MXene flakes in the solution was calculated for two sizes: 1300 × 900 and 500 × 350 nm. Concentrations for both cases were taken as 100 μg/mL. Density of Ti_3_C_2_ −4.2 g/cm^3^.^31^ The thickness of a Ti_3_C_2_T_*x*_ flake was taken as 1.15 nm, which is in the range of 1.14–1.18 nm thickness for single Ti_3_C_2_T_*x*_ flake according to the literature.^32^ Volume (*V*) of a single MXene Ti_3_C_2_T_*x*_ flake is

V=l·w·h

where *l* and *w* are the lengths of the sides, *h*—thickness of a MXene flake.

For 1300 × 900 nm flakes: *V*_1_ = 1.35 × 10^–15^ cm^3^ and for 500 × 350 nm flakes: *V*_2_ = 2.01 × 10^–16^ cm^3^. The weight (*m*) of each MXene flake

m=ρ·V

where ρ is density of Ti_3_C_2_ MXene. For 1300 × 900 nm flakes, the weight of a single flake is *m*_1_ = 5.65 × 10^–15^g, and for 500 × 350 nm flakes - *m*_2_ = 8.45 × 10^–16^g. The number of Ti_3_C_2_ MXene flakes in 100 μg was calculated as *N*_1_ = 1.77 × 10^10^ for 1300 × 900 nm flakes and *N*_2_ = 1.18 × 10^11^ for 500 × 350 nm flakes.

The average volume (*V*_av_) of water per each flake in 100 μg/mL solution was calculated from the formula

$$V_{av}=\frac{1}{N}$$

*V*_av1_ = 5.65 × 10^–11^ mL or 56.51 μm^3^ for 1300 × 900 nm flakes and *V*_av2_ = 8.45 × 10^–12^ mL or 8.45 μm^3^ for 500 × 350 nm flakes.

The distance between MXene flakes can be variable because the orientation of the flakes is random. Here, we calculate maximum (*d*_max_) and minimum (*d*_min_) distances corresponding to parallel and in-plane orientation of flakes as depicted in Supporting Information Figure S3a,b, respectively. Accordingly, MXene flakes could be differently oriented toward bacteria, as it is shown in Supporting Information Figures S3c,d. Supporting Information Figure S3d also illustrates the space that MXene flakes could occupy via variation of orientation.

The maximum distances (*d*_max_) between geometrical centers of Ti_3_C_2_ MXene flakes when flakes are parallel to each other (Figure S3a) correspond to side of a cube with volume *V*_av_

$$d_{\max1}\approx \sqrt[{3}]{V_{av1}}=\sqrt[{3}]{56.51}=3.84\;\mu m$$

for 1300 × 900 nm flakes and

$d_{\max2}\approx \sqrt[{3}]{V_{av2}}=\sqrt[{3}]{8.45}=2.04\;\mu m$ for 500 × 350 nm flakes.

The minimum distance between edges of flakes when they are oriented in-plane is calculated as side length of MXene flake subtracted from maximum distance between centers of flakes

$$d_{\min}=d_{\max}-l$$

*d*_min1_ was calculated to be 2.54 μm for 1300 × 900 nm MXene flakes and *d*_min2_ −1.54 μm for 500 × 350 nm flakes.

To calculate the number of MXene flakes per each bacterium, we considered that a milliliter of medium contains *B* ≈ 10^6^ bacteria. Amount of Ti_3_C_2_ MXene flakes per bacterium (Nb) is

$$Nb_{1}=\frac{N_{1}}{B}=\frac{6.76\cdot 10^{9}}{10^{6}}=17700$$

for 1300 × 900 nm MXene flakes and

$$Nb_{2}=\frac{N_{2}}{B}=\frac{45.4\cdot 10^{9}}{10^{6}}=118000$$

for 500 × 350 nm MXene flakes.

### 2.10 MXene-Based Photothermal Ablation

The antibacterial measurements were conducted using bacterial suspensions of *E. coli* at a concentration of 10^6^ CFU/mL. The bacteria were cultured on Mueller–Hinton Agar (MHA) and incubated at 37 °C for 24 h.

For the antibacterial testing, 200 μL aliquots of the prepared bacterial suspensions were added in triplicate to sterile microplates (Sarstedt, Germany) and inoculated with Ti_3_C_2_T_*x*_ MXene. The microplates were then incubated at 37 °C for 4 h.

In a separate experiment, 100 μL of bacterial suspension was added to each well of a sterile 96-well plate, followed by 100 μL of MXene solution (50 μL/mL). The plates were incubated at 37 °C for 6 h, after which they were subjected to continuous mode laser treatment (808 nm, 2 W-10 Hz) using a DEN5A device (Gigaa Optroniks Technology Co., Ltd.). Bacterial cultures pretreated with MXenes were subjected to NIR irradiation for defined time intervals (5, 10, and 15 min). Untreated control wells were included for comparison. Temperature profiles were recorded using an infrared thermal camera (FLIR 55901–2302 T620 High Resolution Infrared Thermal Imaging Camera) over time.

Post-treatment, bacterial viability was assessed using the colony-forming unit (CFU) assay to quantify the effectiveness of PTT. Bacterial viability was assessed at two time points: 30 min and 24 h postirradiation. For each assessment, 10 μL of the bacterial suspension was inoculated onto Mueller–Hinton Agar (MHA) using the streak plate technique. The Petri plates were incubated at 37 °C for 24 h, and colony-forming units (CFU/mL) were quantified (Supporting Information, Figure S4).

For SEM detection of the MXene-based photothermal ablation effect, glass samples (0.2 × 0.3 cm) were incubated with a bacterial suspension according to the previously described protocol to obtain surfaces covered with biofilm. The samples were then treated with a laser under previously described conditions. After treatment, the samples were washed with phosphate-buffered saline (PBS, pH 7.4) and fixed twice in a 1% glutaraldehyde solution in PBS for 15 min each. Subsequently, the samples were washed twice with PBS for 15 min. Dehydration was performed using a graded ethanol series (25%, 50%, 80%, and 96%). For SEM imaging, the dried samples were mounted on carbon tape and coated with a 5 nm layer of gold by sputtering.

### 2.11 Targeted MXene-Based Photothermal Microbial Ablation

The preparation of Ti_3_C_2_T_*x*_-PDA complexes and antibody (Ab) binding was performed with modifications to the method described in our previous research.^33^ Briefly, an aqueous Ti_3_C_2_T_*x*_ solution (1 mg/mL, 1 mL) was mixed with tris buffer (0.01 M, 30 mL, pH ≈ 8.5; CAS 77-86-1, Sigma-Aldrich) and sonicated for 10 min. After sonication, dopamine hydrochloride (110 mg; CAS 62-31-7, Sigma-Aldrich) was added to the solution and stirred for 2 h. The resulting Ti_3_C_2_T_*x*_-PDA complexes were collected via centrifugation at 5000 rpm for 8 min, followed by rinsing with Milli-Q water.

For anti-*E. coli* polyclonal antibody (ABIN3027591, antibodies-online.com, US) immobilization, 100 μL of a 0.1% BSA/Ab solution (1.6 mg/mL) was added to 1 mL of the Ti_3_C_2_T_*x*_-PDA complex in 0.1% NaN_3_ (CAS 26628-22-8, Sigma-Aldrich)/PBS. The mixture was incubated overnight on a shaker at 350 rpm. After incubation, the solution was washed three times with 0.1% NaN_3_/PBS (pH 7.4), ensuring that the pH did not exceed 8 to prevent PDA degradation. The resulting Ti_3_C_2_T_*x*_-PDA-Ab complexes were collected by centrifugation at 3500 rpm for 10 min.

Biofilms were established by inoculating cultures of *E. coli* or *S. aureus* in nutrient broth at a concentration of 10^8^ CFU/mL (100 μg/well) and incubating for 24 h to ensure adequate biofilm maturation. After biofilm formation, the wells were washed twice with PBS to remove nonadherent bacteria. Ti_3_C_2_T_*x*_ MXene was then applied directly to the biofilm at a concentration of 50 μg/mL. After 2 h of coincubation, the materials were removed from the wells using PBS washes before irradiation. This step allowed for the selective binding of Ti_3_C_2_T_*x*_-PDA-Ab complexes to *E. coli*, demonstrating their targeted interaction and potential for therapeutic applications. The *E. coli* and *S. aureus* biofilms exposed to Ti_3_C_2_T_*x*_-PDA-Ab complexes were subjected to laser irradiation at 4 W, 50 Hz, for 10 min.

For the Resazurin viability assay, a 10% v/v solution of commercially available resazurin (Sigma-Aldrich, USA) was added to each well. Bacterial viability was assessed based on the color change of resazurin from blue/purple to fluorescent pink. The bacteria were incubated at 37 °C for 2 h, until the color change was visible.

To distinguish between bacteriostatic and bactericidal effects of MXene-based photothermal therapy, post-treatment bacterial regrowth was assessed. After laser exposure, treated and untreated wells were incubated at 37 °C for 24 h to allow potential surviving bacteria to proliferate. Following incubation, 25 μL of bacterial suspension from each well was plated onto Mueller–Hinton Agar (MHA) and incubated for another 24 h at 37 °C. The colony-forming units (CFU/mL) were then quantified to determine the extent of bacterial survival.

Following this, the wells were washed twice with PBS and subjected to fluorescence Live/Dead staining. The broth was discarded, and all wells were washed with PBS. A 200 μL aliquot of Calcein AM working solution (20 μL Calcein AM + 980 μL PBS) was added to each well and incubated for 1 h at room temperature, protected from light. After incubation, the Calcein AM solution was discarded, and the wells were washed with PBS. Then, 200 μL of PI working solution (100 μL PI + 1 mL PBS) was added to each well and incubated for 6 min. The PI solution was discarded, the wells were washed with PBS, and 100 μL of fresh PBS was added to each well. Finally, the results were observed using an inverted fluorescent microscope.

### 2.12 Photothermal Microbial Ablation Using MXenes in an Animal Wound Model

Bacterial strains of *S. aureus* (B 918) and *E. coli* (B 926) were obtained from the Ukrainian Collection of Microorganisms (UCM) at the D.K. Zabolotny Institute of Microbiology and Virology, National Academy of Sciences of Ukraine (IMV NASU, Kyiv, Ukraine), and *Pseudomonas aeruginosa* (ATCC 27853, Manassas, VA, USA). All media (Muller–Hinton agar (#70191), Muller–Hinton broth (#70192), MacConkey agar #70143, mannitol-salt agar #63567, and cetrimide agar #1.05284 Merck Millipore) were purchased from Sigma-Aldrich (Darmstadt, Germany) and used without further modification.

#### 2.12.1 Cell Viability and Biocompatibility under Photothermal Therapy (PTT) In Vitro

To assess the impact of laser radiation on normal tissue, primary human dermal fibroblasts (HDFs) were obtained from a healthy donor who provided informed consent for the use of their skin tissue in scientific research. The cells were isolated from the dermis using enzymatic digestion with a 0.25% trypsin solution and cultured in Dulbecco’s Modified Eagle Medium (DMEM) supplemented with 10% fetal bovine serum (FBS), 100 U/mL penicillin, 100 U/mL streptomycin, and 0.25 mg/mL amphotericin B (complete medium). Cultures were maintained at 37 °C in a humidified incubator with 5% CO_2_ until they reached 70–80% confluence, after which they were used for experiments. HDFs at passage 6 were used in this study. The HDFs were exposed to near-infrared (NIR) laser irradiation using an EVOLINE-1 laser (Minsk, Republic of Belarus) with a wavelength of 755 nm, pulse duration of 8 ms, energy fluence of 10 J/cm^2^, and frequency of 1.0 Hz. Irradiation durations were varied as follows: 10 + 10 + 10 s, 20 s, 15 s, and 5 + 5+5 s. To ensure uniform exposure, the laser was applied perpendicularly to the cell monolayer at a fixed distance of 3 cm. Control groups consisted of nonirradiated fibroblasts cultured under identical conditions. Following laser treatment, cells were analyzed for morphological changes and viability.

Cell viability was assessed using a resazurin-based luminescence assay, with emission at 560 nm and excitation at 590 nm, measured using the Varioskan LUX multimode microplate reader.

Cell morphology was evaluated through DAPI staining. Samples were incubated with DAPI for 30 min, then washed three times with PBS. Stained cells were visualized using an inverted fluorescence microscope (ECLIPSE Ti2-E), and images were analyzed using specialized software.

#### 2.12.2 Antibacterial Activity of Photothermal Therapy (PTT) In Vitro

The experiment utilized bacterial strains of *S. aureus* (B 918), *E. coli* (B 926), and *P. aeruginosa* (ATCC 27853). For the experiments, freshly grown cultures were diluted in Mueller–Hinton medium to a concentration of 10^6^ cells/mL for individual strains and up to 5 × 10^9^ cells/mL for the mixed bacterial culture. MXene samples were added to a 24-well plate containing 1 mL of a 1:1 diluted bacterial culture solution, resulting in a final MXene concentration of 50 μg/mL. The wells were then exposed to laser irradiation (755 nm, EVOLINE-1 laser) in the mode of 8 ms, 10 J/cm^2^, and 1.0 Hz. Irradiation durations varied: 10 +10 +10 s, 20 s, 15 s, and 5 +5 +5 s. The samples were divided into four experimental groups based on irradiation time. Viable bacteria were assessed immediately after laser treatment by inoculating 20 μL of bacterial suspension onto nutrient agar plates, followed by incubation at 37 °C for 24 h. Colony-forming units (CFU/mL) were counted and log_10_ transformed.

#### 2.12.3 In Vivo Antibacterial Activity of Photothermal Therapy (PTT)

The experiment involved 12 male laboratory white nonlinear rats (200–240 g) from the vivarium of Sumy State University. The animals were housed in clean plastic cages with stainless steel grates at 22 ± 2 °C, under a 12 h light/dark cycle. They were fed standard pelleted food and provided with drinking water. Animal housing and all procedures were conducted following Directive 2010/63/EU of the European Parliament and the Council. They were approved by the Commission on Bioethics Compliance in Experimental and Clinical Research of Sumy State University (Protocol #2/04 dated 9 April 2024).

MXene (Ti_3_C_2_T_*x*_) was applied to wounds, and laser treatment was performed using an EVOLINE-1 laser (755 nm, 8 ms, 10 J/cm^2^, 1.0 Hz) with an irradiation duration of 10 + 10 + 10 s.

#### 2.12.4 Design of the Animal Experiment

For general anesthesia, intramuscular administration of ketamine in a dosage of 0.05 mL/kg (Ketamine hydrochloride, solution for injection, JSC Farmak, Ukraine) was used. Prior to the operation, the animals’ fur was shaved in the interlobar area, and they were fixed by their limbs on a slide. The surgical field was treated three times with a 70% ethyl alcohol solution and covered with sterile napkins.

A rectangular wound defect measuring 1.0 cm × 1.5 cm was surgically created in the interlobar area of each rat using a sterile scalpel, dissecting through the skin and subcutaneous tissue. The wound edges were fixed with Kocher clamps, and a sterile gauze swab (5 cm × 10 cm), premoistened with a bacterial suspension, was sutured into the wound. The suspension contained daily cultures of *S. aureus* (1.0 mL), *E. coli* (1.0 mL), and *P. aeruginosa* (1.0 mL), each at a concentration of 5 × 10^9^ CFU/mL, suspended in a total of 5 mL of sterile saline. After 72 h, the gauze was removed, and the presence of a purulent wound was confirmed by clinical signs such as skin hyperemia, necrotic tissue debris, and foul-smelling pus. A total of 28 rats were randomly divided into four groups (*n* = 7 each):

(1) purulent wound treated with MXenes and laser irradiation,
(2) purulent wound treated with laser only,
(3) purulent wound without any treatment (control), and
(4) purulent wound treated with Betadine solution.

In group 1, 200 μL of MXenes at a concentration of 100 μg/mL were applied topically to the wound, followed by laser treatment and sterile gauze dressing. Group 2 received laser treatment only. Group 3 received no treatment. Group 4 was treated topically with Betadine solution (Egis Pharmaceuticals PLC, Körmend, Hungary), followed by a sterile dressing. All wound care procedures, including dressing changes, were performed daily under aseptic conditions. Treatment was conducted during both the inflammatory and proliferative phases of wound healing.

#### 2.12.5 Planimetric Examination of Wound Surfaces

Wound defects were photographed daily from day 1 to day 15 using a Canon EOS 600D camera. The area of the wound surfaces was calculated using the open-access ImageJ software, with images taken alongside a standard ruler (1 mm gradation).

### 2.13 Statistical Analysis

All experiments were repeated at least three times. For normally distributed continuous variables, the data are presented as the mean ± standard deviation (SD). One-way analysis of variance (ANOVA) followed by Tukey’s post hoc multiple comparisons test was used when comparing three or more groups. For non-normally distributed data, the Kruskal–Wallis test or the Friedman test was applied. Statistical analysis was performed using GraphPad Prism version 10.3.1. Values of *p* < 0.05 were considered statistically significant.

## 3 Results

### 3.1 Characterization of MXenes

Following the synthesis of MXenes (Figure 1a), a comprehensive characterization of their structural and morphological properties was carried out to confirm their size distribution as well as structural and chemical integrity. Dynamic light scattering (DLS) measurements were performed to determine the size distribution, as shown in Supporting Information Figure S5. In this study, we employed two distinct sizes of MXene flakes (those larger than 1000 nm (large-size) and those smaller than 1000 nm (small-size), based on the understanding that antibacterial properties and cellular interactions can be strongly influenced by the lateral dimensions of 2D nanomaterials.^23^ Based on size, samples were marked as -L (large) and –S (small). Some experiments with Ti_3_C_2_T_*x*_ MXenes were performed using one (large) size, and those MXenes were not marked in the text. Structural characterization was conducted using scanning electron microscopy (SEM), transmission electron microscopy (TEM) with energy-dispersive X-ray spectroscopy (EDX), atomic force microscopy (AFM), and Raman spectroscopy. For detailed structural analysis, representative samples Ti_3_C_2_T_*x*_-S, Nb_2_CT_*x*_-L, V_2_CT_*x*_-S, and Ti_3_CNT_*x*_-L were selected.

**Figure 1:**
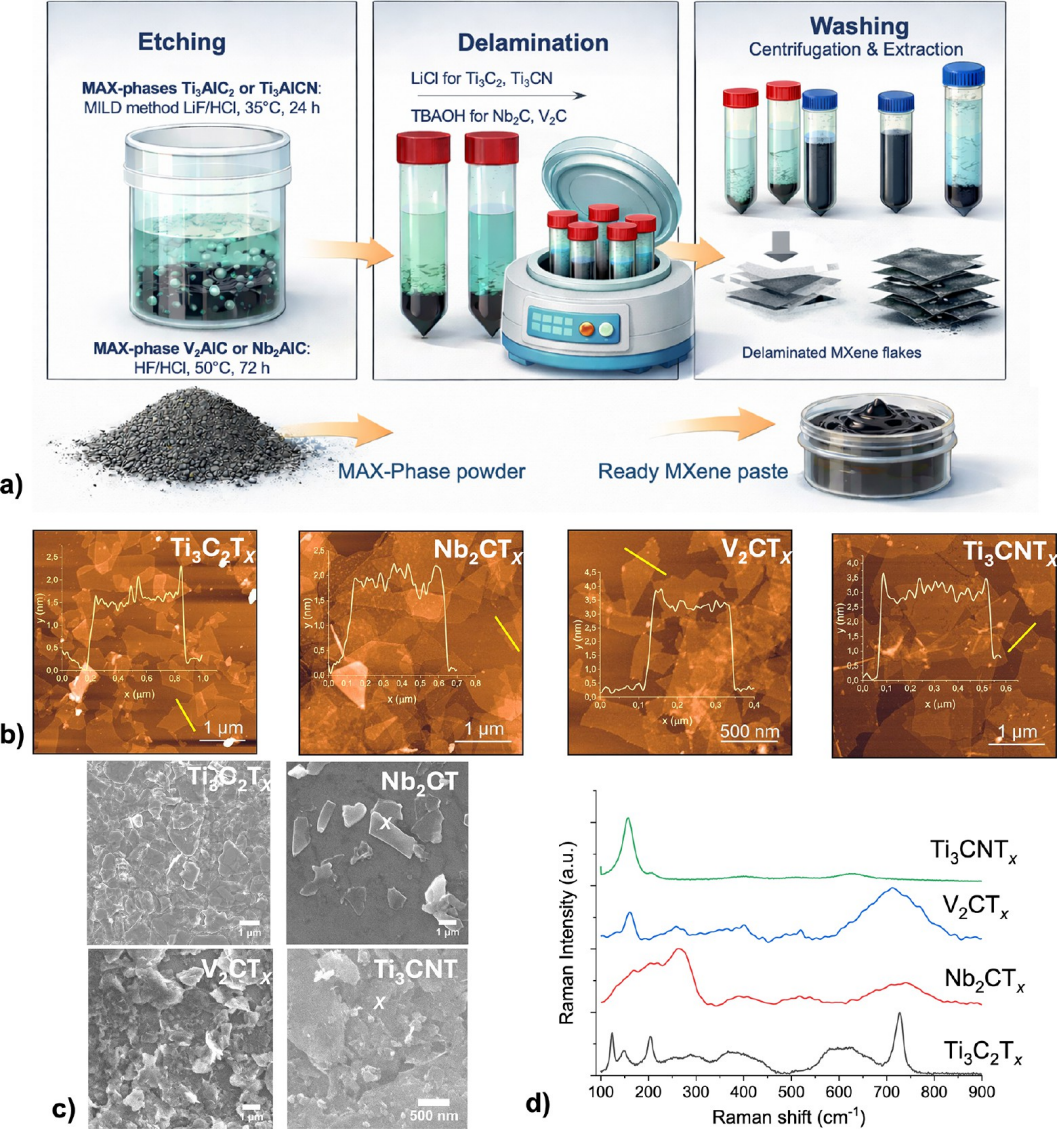
(a) Scheme of MXene synthesis, including etching, delamination and washing steps; (b) AFM images of the synthesized MXene samples (overlaid insets show height profiles of selected MXene flake); (c) SEM images; (d) Raman spectra of MXene samples.

Figure 1b demonstrates the AFM images of MXene with varying lateral sizes across all samples. The average flake dimensions were estimated as follows: 800 ± 300 nm for Ti_3_C_2_T_*x*_, 850 ± 200 nm for Nb_2_CT_*x*_, 500 ± 200 nm for V_2_CT_*x*_, and 1000 nm ± 400 nm for Ti_3_CNT_*x*_. AFM height profiles revealed average thicknesses of 1.5 ± 0.2 nm (Ti_3_C_2_T_*x*_), 2 ± 0.3 nm (Nb_2_CT_*x*_), 3.5 ± 0.3 nm (V_2_CT_*x*_), and 3 ± 0.3 nm (Ti_3_CNT_*x*_), indicating single-layer to few-layer flakes. AFM overestimates the flake thickness due to layers of adsorbed molecules on the substrate and MXene surface. The SEM images (Figure 1c) further support the AFM findings, clearly demonstrating lateral size variation and surface morphology of the MXene flakes. Ti_3_CNT_*x*_ flakes appear significantly larger and more irregular, whereas V_2_CT_*x*_ samples exhibit smaller, fragmented features. The observed lateral size distributions are in good agreement with AFM data and confirmed by TEM/EDX analysis (see Supporting Information, Figure S6).

Raman spectroscopy was employed to analyze the phase composition, surface functional groups, and vibrational modes of the synthesized MXenes (Figure 1d). A 785 nm excitation red diode laser was used, offering enhanced sensitivity to the vibrations of surface species present on the MXene surfaces. The Raman spectrum of Ti_3_C_2_T_*x*_ nanosheets exhibited several characteristic peaks at 123, 148, 204, and 727 cm^–1^, as well as two broad bands near 380 and 600 cm^–1^. The peaks at ∼123 and ∼204 cm^–1^ correspond to in-plane (E_g_) and out-of-plane (A_1g_) vibrations of Ti–C bonds and associated surface terminations (−OH, −O, and −F).^34,35^ The broad features at ∼380 and ∼600 cm^–1^ were most likely due to surface termination vibrations and structural disorder. The 727 cm^–1^ peak is associated with C–C stretching vibrations.^34^ The intensity ratio *I*_123_/*I*_204_ (∼1.09) suggests a strong contribution from surface terminations, while the low *I*_600_/*I*_727_ ratio (∼0.42) points to minimal oxidation and low levels of carbon contamination.

For the Nb_2_CT_*x*_ sample, Raman peaks were observed in the low-frequency region (∼105, 162, 212, and 267 cm^–1^), corresponding to Nb atom vibrations in structures terminated with −OH and =O groups. Specifically, peaks at 105 and 212 cm^–1^ are attributed to Nb–OH (E_g_ and A_1g_), while those at 162 and 267 cm^–1^ arise from Nb =O vibrations. The most intense peak at 267 cm^–1^ (A_1g_) reflects the dominance of oxygen-containing terminations.^35^ Additional peaks at 390, 430, 515, and 681 cm^–1^ are associated with vibrational modes of −OH, −F, and =O groups. A weak band at ∼749 cm^–1^ corresponded to carbon vibrations (E_g_) in Nb_2_C­(OH)_2_, activated by surface-induced symmetry breaking.^36^

The V_2_CT_*x*_ Raman spectrum showed a distinct peak at ∼160 cm^–1^, corresponding to V–C lattice vibrations, along with a broad and intense band at ∼710 cm^–1^. This feature was assigned to either C–C vibrational modes or vibrations of terminal groups in a disordered environment. These observations suggest partial oxidation and the presence of various surface terminations.^35^

For the Ti_3_CNT_*x*_ sample, a main peak was observed at ∼150 cm^–1^, which, upon deconvolution, splits into two components (∼135 and ∼155 cm^–1^). These are attributed to A_1g_-type vibrations of Ti atoms in a mixed C/N bonding environment. The ∼135 cm^–1^ band indicated lattice distortions caused by nitrogen incorporation. A weak peak around 204 cm^–1^ corresponded to vibrations in the Ti–C/N layer and is suppressed due to surface terminations. As noted by Zhang et al.,^37^ compared to Ti_3_C_2_T_*x*_, the Ti_3_CNT_*x*_ spectrum exhibited broader and shifted bands, consistent with a higher degree of disorder and complex bonding environment.^35^ To summarize, the synthesized MXenes used in this study consisted predominantly of single-to few-layered flakes with lateral dimensions varying according to their chemical composition. Structural and chemical analyses confirmed successful delamination, high structural integrity, and minimal presence of toxic halide terminations such as fluorine and chlorine. These characteristics make the selected MXene formulations suitable for further evaluation of their antibacterial properties.

### 3.2 MXene Biocompatibility

To effectively evaluate the antibacterial potential of nanomaterials, it is essential to assess their biocompatibility to identify appropriate, nontoxic dosing ranges. Previous studies have suggested that MXene nanosheets exhibit high biocompatibility, almost regardless of their chemical composition. However, most of these investigations were limited to short-term coincubation periods ranging from 4 to 24 h, which restricts their relevance for long-term biomedical applications.^38,39^ In contrast, our study employed an extended 6 day observation period to assess the prolonged cytotoxic effects of MXenes, providing a more comprehensive evaluation of their long-term biocompatibility. Among the tested MXenes, titanium carbide (Ti_3_C_2_T_*x*_) and titanium carbonitride (Ti_3_CNT_*x*_) demonstrated the highest cell viability over 6 days, indicating superior biocompatibility compared to niobium carbide (Nb_2_CT_*x*_) and vanadium carbide (V_2_CT_*x*_). These trends were consistent in both human keratinocytes (HaCaT) and the melanoma cell line (MaMel 8b), suggesting comparable cytocompatibility profiles across normal and cancerous cell types (Figure 2).

**Figure 2:**
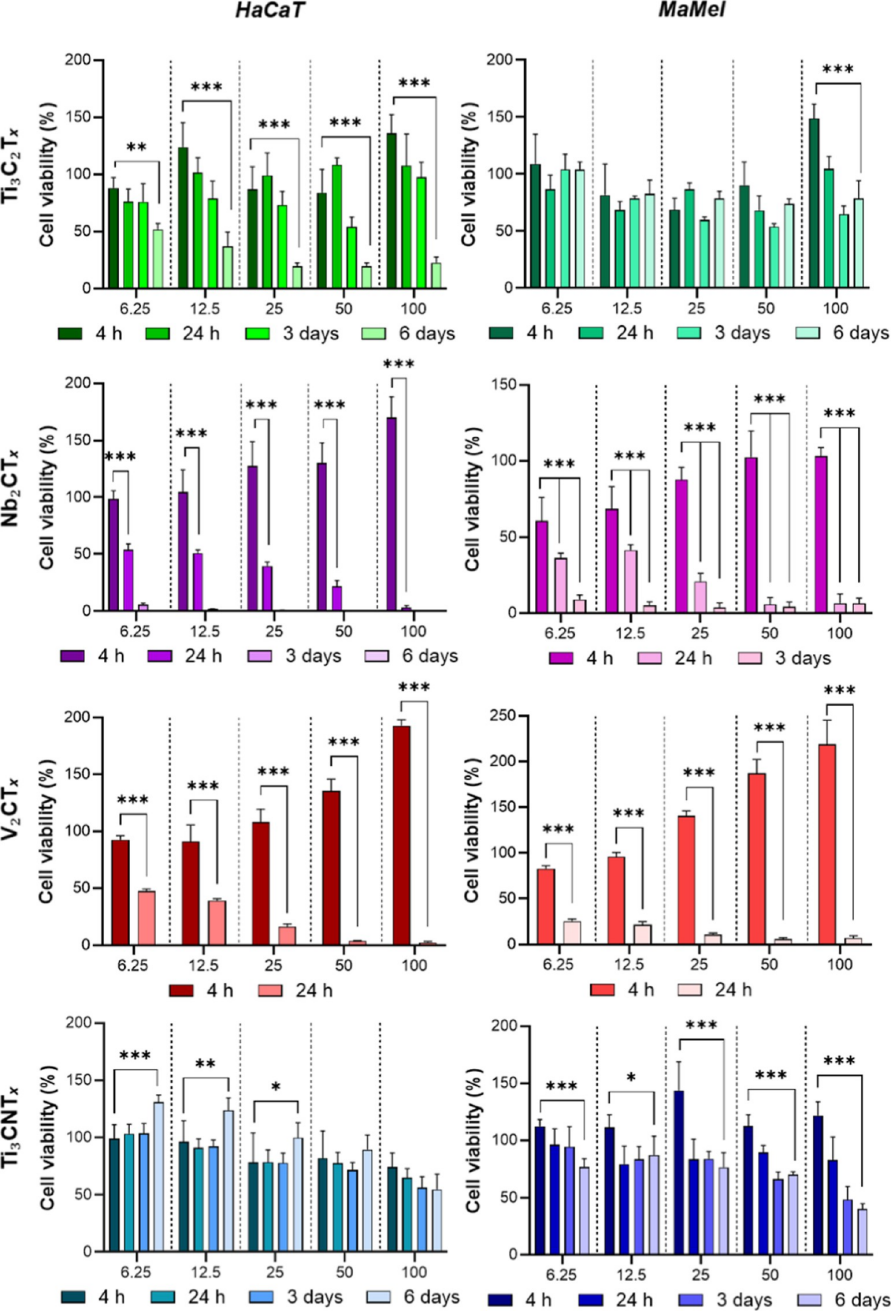
Dose-dependent cytotoxicity of Ti_3_C_2_T_*x*_, V_2_CT_*x*_, Nb_2_CT_*x*_, and Ti_3_CNT_*x*_ MXenes (μg/mL) assessed by resazurin reduction assay over 6 days in HaCaT keratinocytes and MaMel melanoma cells.

V_2_CT_*x*_ was identified as the most cytotoxic material, as no viable cells were detected in either cell line within 24 h of exposure, even at the lowest tested concentration, indicating rapid and complete cytotoxicity. Nb_2_CT_*x*_ nanosheets exhibited moderate toxicity: HaCaT cells showed reduced metabolic activity by day 6, while the MaMel 8b assay was terminated at day 3 due to complete loss of cell viability, highlighting a cell-type-dependent sensitivity. The toxicity of two-dimensional MXene materials such as Nb_2_CT_*x*_ and V_2_CT_*x*_ may be attributed to their rapid oxidative degradation, leading to the formation of cytotoxic niobium- and vanadium-containing oxides, fluoride ions, as well as the release of metal cations; this is particularly relevant for vanadium, which readily forms soluble reaction products with well-documented cytotoxic and genotoxic effects.^40^ In contrast, Ti-based MXenes predominantly oxidize to TiO_2_, a material that is widely regarded as biocompatible and of low toxicity and is routinely used in medical implants and pharmaceutical or food formulations. Accordingly, in our experiments, we did not observe any additional cytotoxicity that could be ascribed to TiO_2_ formation, whereas Nb_2_CT_*x*_ and V_2_CT_*x*_ showed clear toxicity that is consistent with the generation of more harmful oxide and ionic species.

Interestingly, a transient increase in cell viability was observed at the 4 h mark, particularly at higher concentrations. This paradoxical effect may stem from the intrinsic redox activity of MXenes or assay interference due to reactive oxygen species (ROS), which can alter the resazurin reduction reaction and produce artificially elevated readings.

Ti_3_C_2_T_*x*_ nanosheets exposure resulted in a notable increase in MaMel 8b cell viability (with no effect in HaCaT cells) on days 3 and 6 at subtoxic concentrations (6.25 and 12.5 μg/mL), suggesting a potential stimulatory or proliferative effect specific to melanoma cells. Overall, Ti-based MXenes, particularly Ti_3_C_2_T_*x*_ and Ti_3_CNT_*x*_, exhibit favorable cytocompatibility and may hold promise for biomedical applications, owing to their lower cytotoxicity profiles. It should be noted that the in vitro cytotoxicity of MXene nanomaterials can vary significantly depending on the specific cell line used. For example, Ti_3_C_2_ MXene was found to be markedly more toxic to human cancer cell lines (A549 lung carcinoma and A375 melanoma) than to noncancerous cells (MRC-5 lung fibroblasts and HaCaT keratinocytes).^23^ Similarly, another study reported higher cytotoxic effects of Ti_3_C_2_T_*x*_ nanosheets on HeLa cervical cancer cells compared to normal fibroblasts.^41^ These findings indicate that different cell types exhibit distinct tolerance levels to MXene exposure, resulting in cell line–dependent variations in cytotoxic outcomes.

### 3.3 MXenes’ Antibacterial Activity

In this study, we employed multiple complementary methods to assess the antibacterial activity of MXene nanosheets, taking into account their chemical composition and flake size. The disk diffusion method (Supporting Information, Figure S7a) was initially selected due to its widespread use as a standard screening technique for antibiotics and antimicrobial agents. Although previous studies have reported inhibition zones for MXenes and their composites,^42^ this method primarily provides a qualitative measure of antimicrobial potential. Importantly, the disk diffusion assay relies on the ability of compounds to diffuse through agar, which may not be suitable for MXenes due to their relatively large flake size, poor solubility, and limited diffusion mobility without applying an electric field. As a result, compounds that do not readily diffuse, such as 2D materials, may yield false-negative outcomes. In addition, variability in molecular weight, solubility, and charge of surface terminations can further affect diffusion and lead to inconsistent results, particularly when compared to small molecules or metallic nanoparticles such as silver.

Consistent with these limitations, none of the tested MXene nanosheets exhibited visible zones of inhibition in the disk diffusion assay. To further validate this observation, we also employed the drop dilution method, in which concentrated MXene suspensions were applied directly onto bacterial cultures. However, no antimicrobial activity was observed using this method either, suggesting that under the tested conditions, MXene suspensions do not exhibit significant antibacterial effects (Supporting Information, Figure S7b). It should be noted that we used an exceptionally high concentration of MXenes (2000 μg/mL), which exceeds the concentrations typically reported in similar studies. The lack of antibacterial activity even at such elevated doses underscores the limited efficacy of these materials in suspension-based antibacterial applications, at least under the tested conditions.

It is important to note that the agar diffusion assay may lack the sensitivity required to detect weak antimicrobial effects, particularly at low concentrations. Compared to the microdilution method, this assay requires a relatively large quantity of the test substance, which can be impractical. Moreover, the limited diffusion capacity of two-dimensional materials like MXenes further reduces the effectiveness of this method.

To accurately determine the minimum inhibitory concentration (MIC) and minimum bactericidal concentration (MBC) of the MXenes, we employed the broth microdilution assay. However, we observed that during the dispersion of MXenes in the nutrient broth, a significant agglomeration occurred. This is likely due to the formation of a protein corona in the protein-rich media, which reduces colloidal stability and leads to flake aggregation. Notably, the degree of agglomeration increased with higher MXene concentrations, complicating the visual determination of MIC values.

Despite testing at high concentrations (up to 1000 μg/mL), low antibacterial activity was observed across all tested MXenes against both Gram-positive (*S. aureus*) and Gram-negative (*E. coli*) bacteria (Figure 3). Among the studied materials, V_2_CT_*x*_ and Nb_2_CT_*x*_ showed a stronger antibacterial effect compared to Ti-based MXenes. Additionally, small-sized MXene flakes demonstrated enhanced antibacterial potential, likely due to their ability to penetrate bacterial membranes more effectively.

**Figure 3:**
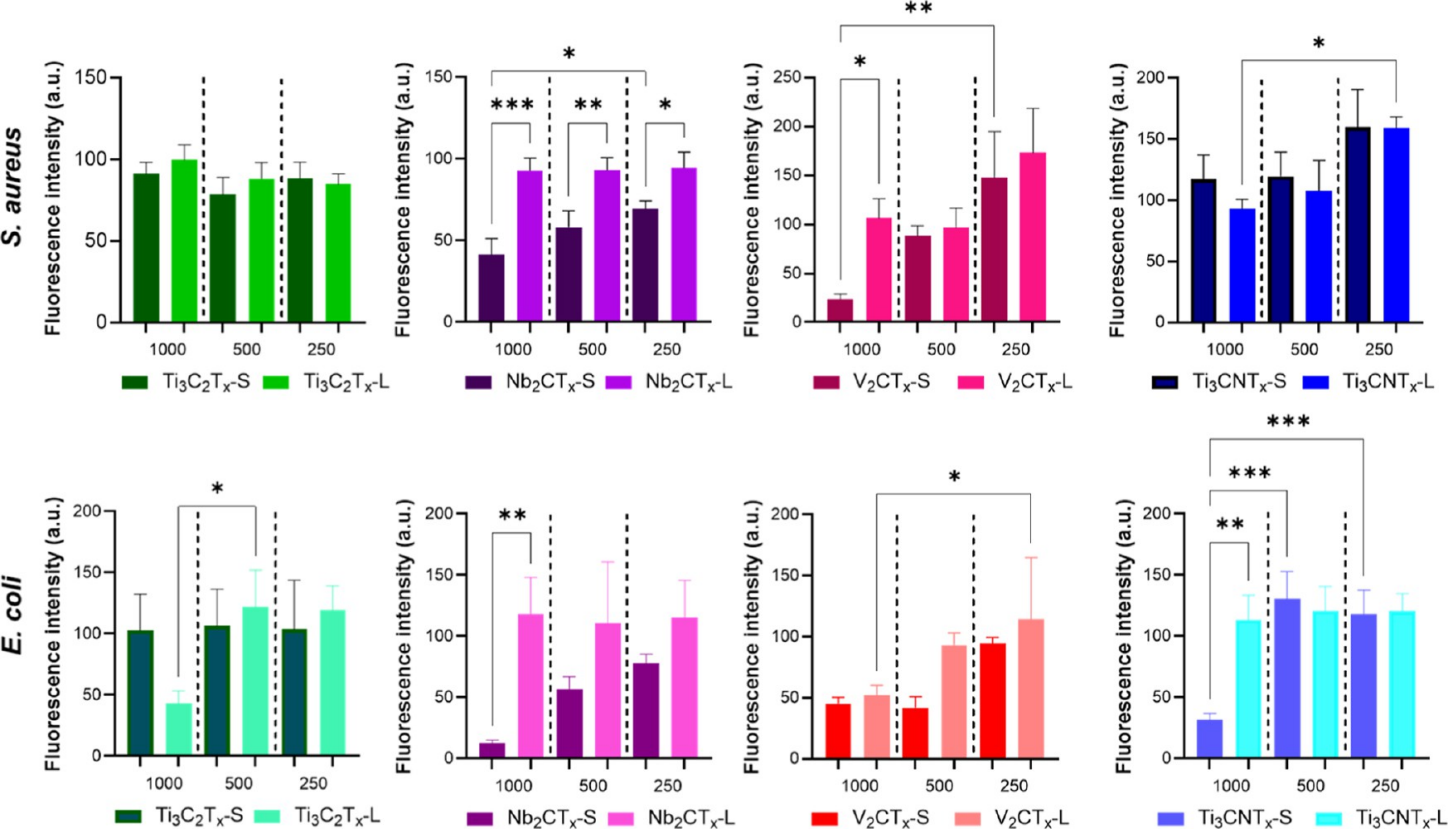
Minimum inhibitory concentration (MIC): comparison of fluorescence emission during the resazurin assay, showing the growth of *S. aureus* and *E. coli* exposed to different concentrations (μg/mL) of MXene.

Nevertheless, MIC values for all tested materials were above 1000 μg/mL, and for Ti-based MXenes, exceeded 2000 μg/mL (Supporting Information, Figure S8). Due to sample turbidity and aggregation, MICs could not be determined visually with sufficient accuracy. Therefore, we proceeded with MBC determination by subculturing from wells onto agar plates. The MBC values also exceeded the highest concentrations (2000 μg/mL) tested for most samples, although partial bactericidal activity was observed in small-sized V_2_CT_*x*_ and Nb_2_CT_*x*_ variants (Supporting Information, Figure S9a,b).

As demonstrated by both the disk diffusion and broth microdilution assays, all tested MXene nanosheets exhibited low antibacterial activity. Among them, only Nb_2_CT_*x*_ and V_2_CT_*x*_ showed modest antimicrobial effects, which may be attributed to their rapid oxidation, consistent with the results of the cytotoxicity assays (Figure 2). To enable a more detailed investigation of antibacterial mechanisms while minimizing potential confounding effects from oxidative degradation, subsequent experiments focused exclusively on Ti_3_C_2_T_*x*_ MXenes, comparing flakes of small and large lateral sizes.

The time-kill kinetics assay evaluates antimicrobial activity by monitoring bacterial growth over time following exposure to varying concentrations of an antimicrobial agent. This method provides valuable insights into whether the antimicrobial effect is time-dependent or concentration-dependent and helps to characterize the dynamic interaction between the agent and the microbial cells.

In this study, the spectrophotometric time-kill assay was conducted at a maximum concentration of 1000–2000 μg/mL, as higher concentrations (≥1000–2000 μg/mL, the MIC range) produced significant turbidity, which interfered with accurate optical density measurements. Notably, the 100 μg/mL dose was chosen based on previous reports demonstrating antibacterial efficacy and favorable biocompatibility—an essential criterion for biomedical applications.^2^

As shown in Figure 4a, the selected concentration (100 μg/mL) failed to inhibit the growth of *S. aureus* and *E. coli*, with bacterial proliferation curves over 24 h comparable to untreated controls. These findings were further supported by bacterial growth on agar plates at all time points (Supporting Information Figure S10a,b). To further validate these results, we performed an automated time-kill kinetics analysis using the HB&L system at concentrations of 25, 50, and 200 μg/mL (Supporting Information Figure S9). The lack of antimicrobial activity was confirmed by both real-time bacterial growth monitoring (Figure 4a) and postincubation agar plating (Figure 4b), both of which showed robust bacterial growth after 24 h at all tested concentrations. Although 200 μg/mL has been previously reported as bactericidal, in our study, turbidity and pigmentation of MXene suspensions at this dose compromised the accuracy of light-scattering-based detection. Nevertheless, the plating results conclusively confirmed the absence of the nanosheets’ bactericidal activity, even at this higher concentration.

**Figure 4:**
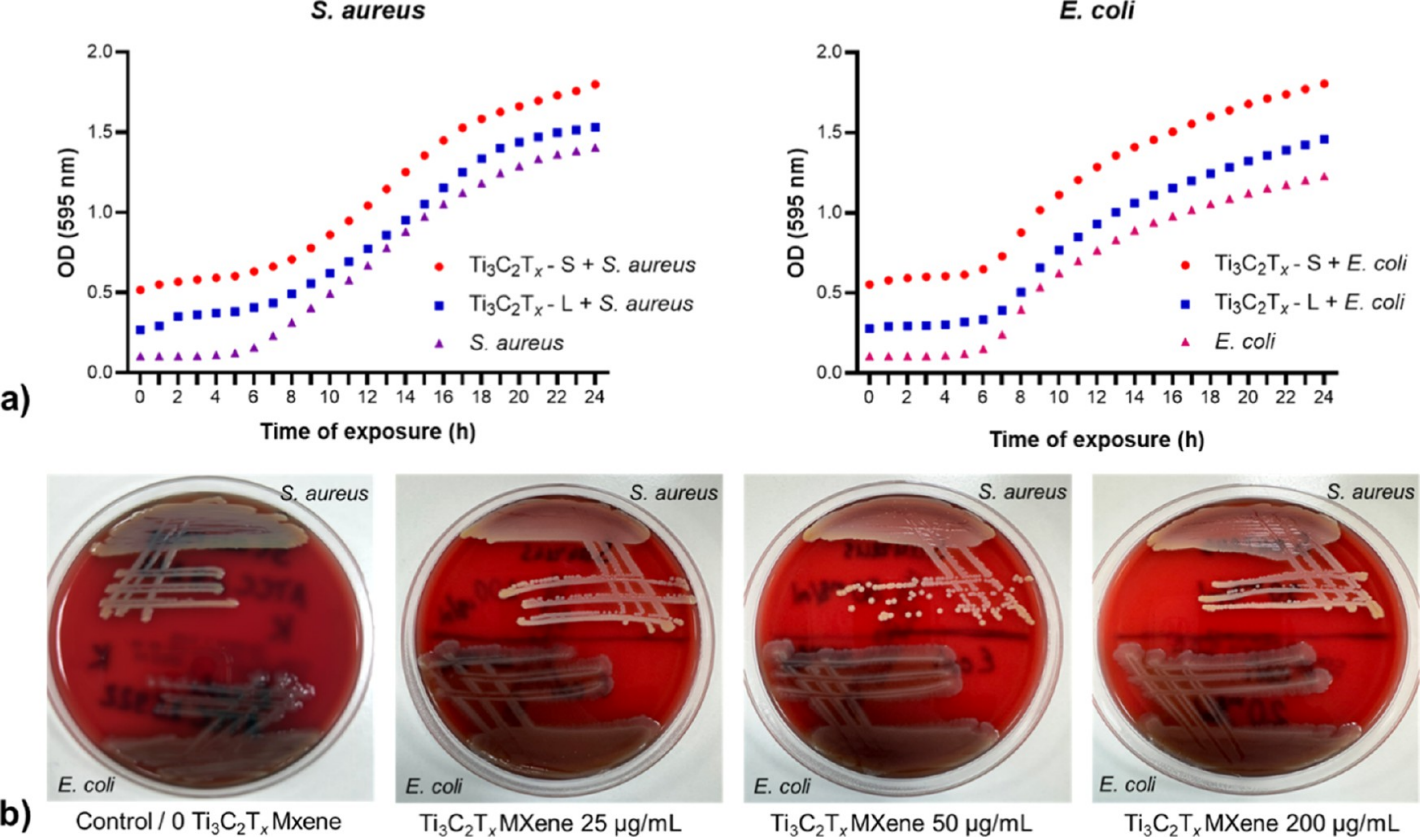
Bacterial growth dynamics in response to 100 μg/mL Ti_3_C_2_T_*x*_ MXene treatment during 24 h. Comparative growth of *S. aureus* and *E. coli* in the presence of different concentrations of MXene: HB&L Nephelometry (a) and plate count results (b).

Several studies have demonstrated that Ti_3_C_2_T_*x*_ MXenes, often modified or combined with other materials (e.g., chitosan or polypropylene), exhibit antibiofilm properties without the need for phototherapy or additional catalytic agents. For example, Ti_3_C_2_T_*x*_ MXene incorporated with chitosan showed bactericidal effects through physical disruption and direct contact with bacterial cells, without any light activation or metallic additives.^43^ Similarly, polypropylene fabrics coated with Ti_3_C_2_T_*x*_ MXene flakes exhibited strong antibacterial and antibiofilm activity via a “nanoblade” effect and antiadhesion mechanism, again without relying on photothermal activation or ROS-generating materials.^42,44^ Additionally, Ti_3_C_2_T_*x*_-coated PVDF membranes effectively inhibited bacterial adhesion and biofilm formation through passive surface properties, with no external stimuli applied.^7^

In this study, we evaluated the ability of pristine Ti_3_C_2_T_*x*_ MXene nanosheets (without any modifications) as a model material to determine their true antibiofilm capabilities by cultivating bacterial cells on polystyrene microtiter plates in the presence of MXene suspensions. Our results demonstrated that Ti_3_C_2_T_*x*_ exhibited antibiofilm activity only at high concentrations, with a noticeable reduction in biofilm viability observed only at 2000 μg/mL, where viability dropped below 50% that is not enough for combating biofilm growth (Figure 5). Compared to planktonic bacterial inhibition assessed via the microdilution assay, the biofilm model revealed a more pronounced antibacterial effect for large-sized MXenes. This might be attributed to the greater surface area of larger flakes, which could enhance physical interaction with bacterial colonies and interfere more effectively with biofilm integrity.

**Figure 5:**
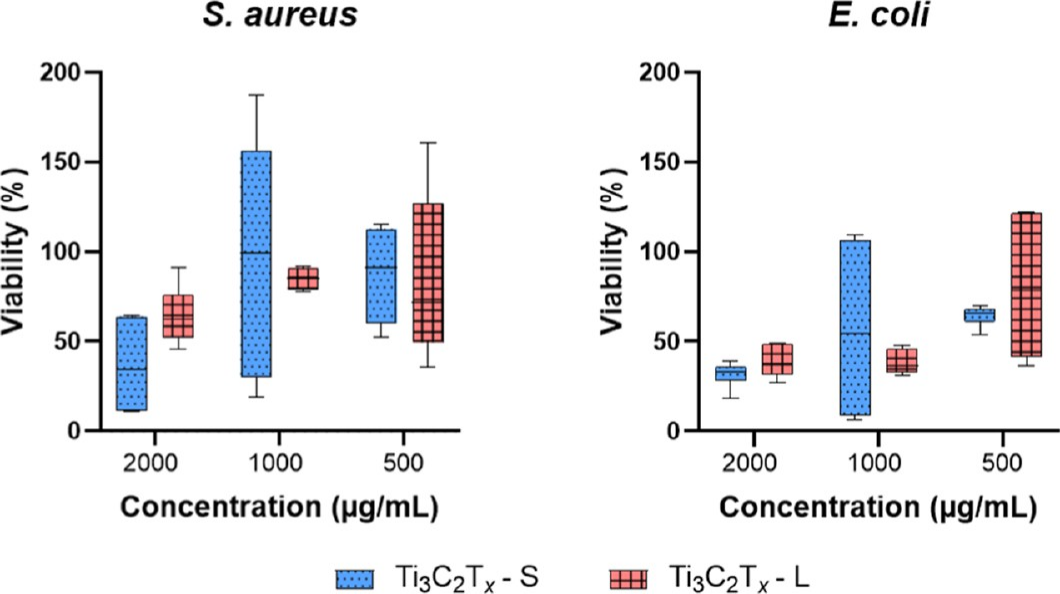
Crystal violet biofilm assay: graphical representation of remaining biofilm mass (%) exposed to Ti_3_C_2_T_*x*_ MXene 24 h incubation.

Interestingly, the apparent antibiofilm activity of large-sized MXenes may also reflect mechanical interference rather than a direct bactericidal effect. During the 24 h incubation, MXene particles tended to sediment at the bottom of the well despite continuous shaking. This sedimentation could physically obstruct bacterial attachment and biofilm development on the surface, leading to a concentration-dependent antibiofilm effect unrelated to classical antimicrobial mechanisms such as ROS generation or membrane disruption.

### 3.4 Validation of MXene’s Antibacterial Mechanisms

In our study, we did not observe strong antibacterial activity from MXenes, particularly at subtoxic concentrations. This highlights the need to critically evaluate the primary antibacterial mechanisms reported in previous studies—namely, ROS-mediated damage and mechanical disruption via the “nano-knife” effect.

MXenes have been shown to exhibit the potential for ROS generation, often linked to antibacterial effects. Their ability to catalyze Fenton-like reactions, similar to Fe-based systems, is thought to arise from the redox cycling of their multivalent metal centers, leading to the production of highly reactive hydroxyl radicals (−OH). Upon coincubation with bacteria, we observed a rapid increase in ROS levels within 5 min, peaking between 15 and 30 min, followed by a significant decline by 90 min (Figure 6). This suggests a transient ROS burst following MXene exposure.

**Figure 6:**
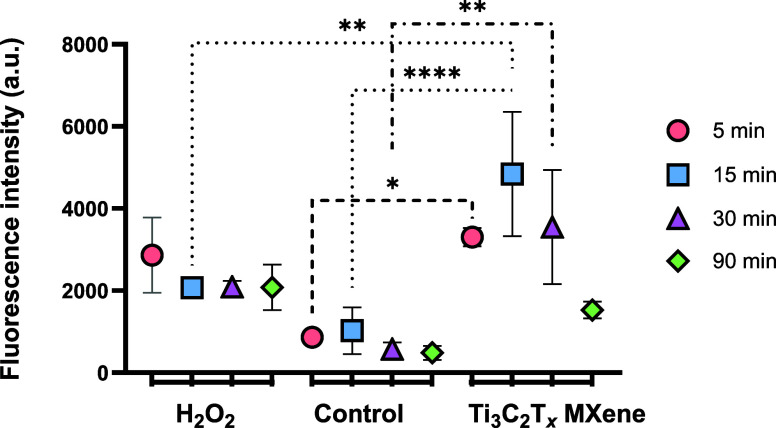
ROS expression in bacteria exposed to Ti_3_C_2_T_*x*_ MXene over time under different experimental conditions. Control represents untreated bacterial suspensions.

MXene nanosheets are known to produce a variety of ROS species—hydroxyl radicals, superoxide anions, hydrogen peroxide, and singlet oxygen—during their interaction with bacterial and mammalian cells.^2^ However, our findings indicate that the magnitude and duration of ROS production were insufficient to elicit a sustained antibacterial effect. One possible explanation is that bacteria possess robust antioxidant defense systems, including enzymes such as catalase and superoxide dismutase, which neutralize ROS and mitigate oxidative damage. Furthermore, ROS-mediated bacterial killing is often dose- and time-dependent, suggesting that higher MXene concentrations or extended exposure may be required to achieve significant antimicrobial activity. Notably, we also observed that ROS levels significantly decreased at later time points (90 min), most likely because MXenes themselves can scavenge reactive oxygen species.^17,45^ Their surface functional groups, electron transfer activity, high surface area, and redox behavior enable MXenes to quench reactive species, potentially protecting both bacterial and mammalian cells from prolonged oxidative stress. Although MXenes generate an initial burst of ROS, this surge was short-lived. It did not sustain antibacterial activity over extended incubation, limiting their effectiveness as ROS-driven antimicrobial agents under the conditions tested.

A widely accepted interpretation of MXene antibacterial activity is their function as “nanoknives”, whereby the sharp edges of 2D flakes penetrate bacterial membranes, causing physical disruption and cell death upon direct contact or surface attachment.^17^ To investigate this mechanism, we performed SEM analysis following incubation of *S. aureus* and *E. coli* with Ti_3_C_2_T_*x*_, aiming to assess whether membrane rupture occurs due to mechanical damage. Our results revealed no evidence of membrane disruption in either bacterial species (Figure 7). Instead, the MXene nanosheets were observed to adhere to or coat the bacterial surfaces. Their localization pattern was size-dependent: smaller flakes adhered to the membrane, while larger sheets appeared to envelop the bacterial cells (Supporting Information, Figure S11). TEM further confirmed the structural integrity of the bacterial membranes, while clearly showing the presence of MXene material on their surfaces (Supporting Information, Figure S12). EDX analysis identified titanium elements corresponding to Ti_3_C_2_T_*x*_, supporting the nanosheet localization observed in TEM. These findings suggest that, under the tested conditions, mechanical damage by MXene edges does not occur, challenging the “nanoknife” hypothesis in this context.

**Figure 7:**
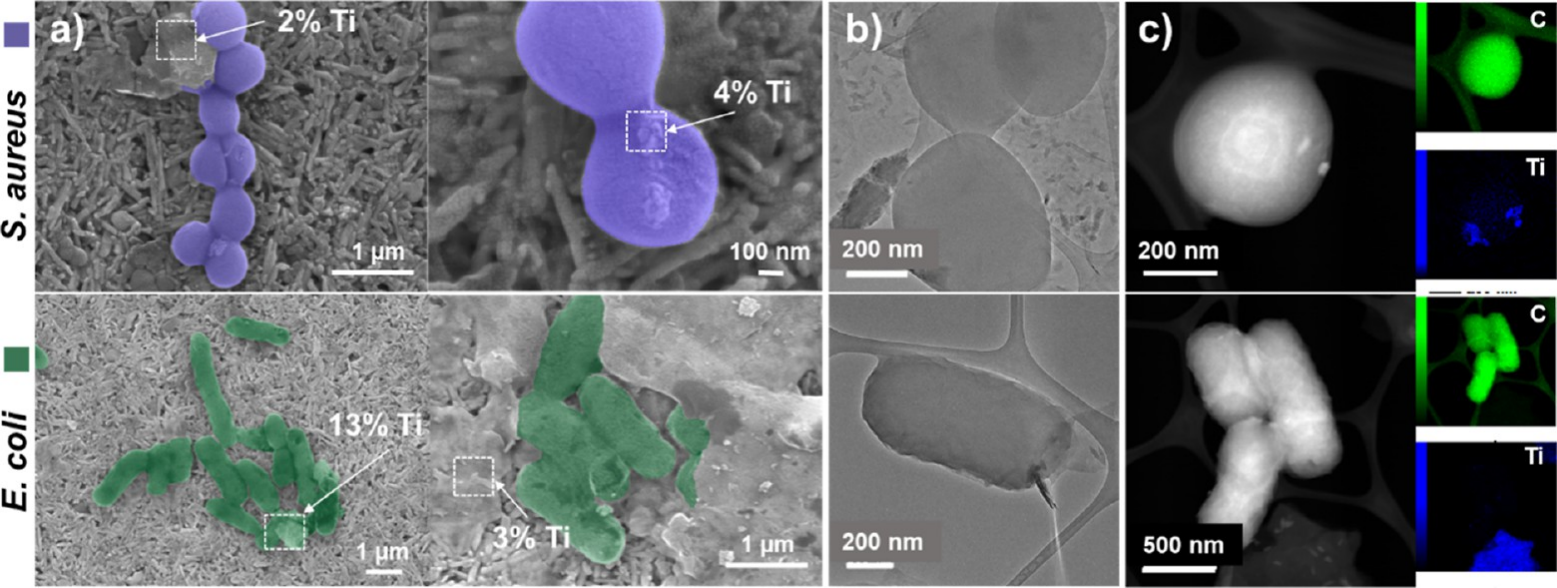
(a) SEM images of the *S. aureus* (top panel) and *E. coli* (bottom panel) treated with 2000 μg/mL of Ti_3_C_2_T_*x*_ MXene, at low and high magnification, respectively (bacteria were artificially colored for easy recognition). The squares indicate the areas of EDX analysis for determining the presence of Ti (at%). (b) TEM images of the *S. aureus* (top panel) and *E. coli* (bottom panel) treated with 2000 μg/mL of Ti_3_C_2_T_*x*_ with (c) EDX maps of elemental distribution of carbon (C) and titanium (Ti).

To minimize MXene nanosheets sedimentation and ensure uniform dispersion during incubation, we investigated the potential contribution of the “nanoknife” mechanism by coincubating *S. aureus* and *E. coli* with Ti-based MXenes under continuous rotational conditions (Supporting Information, Figure S1a,b). Maintaining homogeneous suspension was critical to maximizing the probability of direct contact between bacterial membranes and MXene surfaces, a prerequisite for physical membrane disruption via sharp edges. Moreover, preventing sedimentation helps preserve the bioactive surface area of 2D materials throughout extended exposure. Under these conditions, MXenes remained evenly distributed in solution, and comparative analysis of bacterial growth was performed against static control cultures. Despite improved dispersion and extended contact, no significant difference in colony formation was observed between MXene-treated and untreated groups for either *S. aureus* or *E. coli*, indicating limited or absent antibacterial activity under the tested conditions (Supporting Information, Figure S1c).

To summarize, no ROS-mediated or “nano-knife” antibacterial mechanisms of MXene nanosheets were validated in our study. The short-lived ROS burst observed during the first hour after exposure was insufficient to provide a continuous antibacterial effect, and MXene interaction did not lead to bacterial membrane rupture. These findings challenge the widely proposed ROS and “nano-knife” mechanisms of MXene antibacterial action, at least under the tested conditions and concentrations. They highlight the need for reevaluation of MXene–bacteria interaction models, particularly at subtoxic doses relevant for biomedical applications.

### 3.5 Photothermal Bacteria Ablation

In contrast to direct coincubation of MXenes with bacterial cells, photothermal therapy (PTT) offers a promising strategy for MXene-mediated bacterial eradication. Although PTT is well-established as an anticancer treatment, growing evidence highlights its potential antibacterial applications. For instance, studies show that under dark conditions, there is no significant difference in colony numbers between control and treated groups, indicating no antimicrobial activity without light. However, under light irradiation, the antibacterial efficacy of Ti_3_C_2_, Ag_2_S, and Ag_2_S/Ti_3_C_2_ hybrids is significantly enhanced due to photoexcitation.^46^ Additionally, Cu_2_O/Ti_3_C_2_T_*x*_ hybrids combine photothermal effects with catalytic reactive oxygen species (ROS) production to achieve potent antimicrobial performance.^47^ Moreover, an MXene-PVA/metformin nanoplatform has demonstrated strong inhibition of biofilm formation through NIR-triggered photothermal therapy and immune activation, validated both in vitro and in an MRSA biofilm infection mouse model.^48^ When exposed to near-infrared (NIR) laser irradiation, photothermal agents like MXenes absorb light via surface plasmon resonance, converting it into heat. This localized temperature elevation disrupts bacterial membranes, denatures proteins, induces protein leakage, and ultimately results in irreversible microbial cell death.

In this study, we used Ti_3_C_2_T_*x*_ MXenes at a concentration of 50 μg/mL, a dose verified as biocompatible based on cell viability data (Figure 2). Samples were irradiated with NIR for 5, 10, and 15 min. As shown in Figure 8a, a significant antibacterial effect was observed after just 5 min of irradiation, with complete bacterial eradication following 10 and 15 min of treatment (Figure 8a).

**Figure 8:**
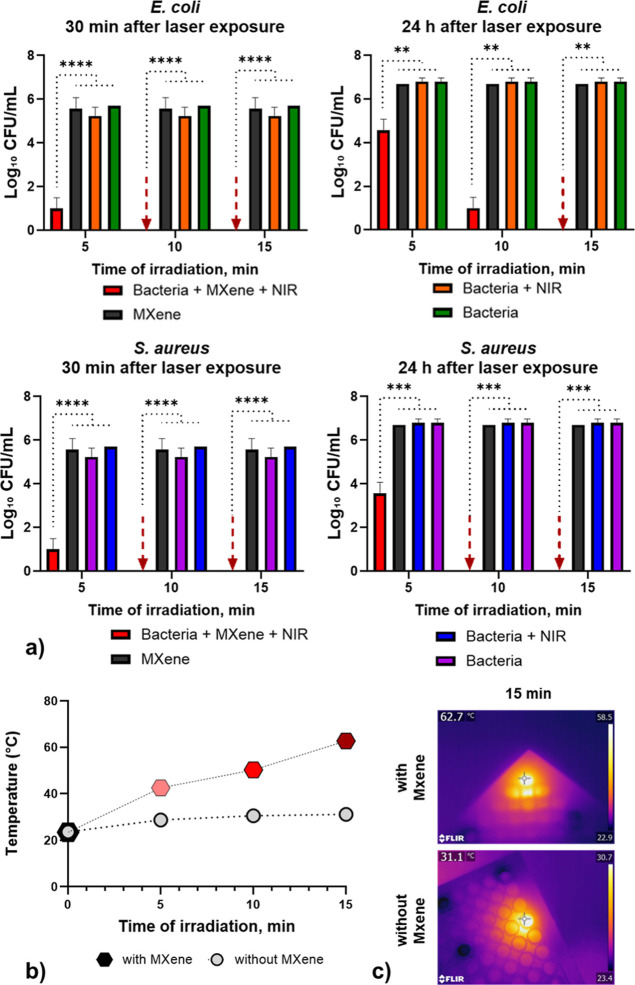
Time-dependent antibacterial efficacy of Ti_3_C_2_T_*x*_ MXene after different irradiation exposure times, with (a) quantitative analysis of antibacterial activity, (b) average temperature measurements after laser exposure at different time points, (c) representative thermal imaging of samples after 15 min of laser irradiation.

Temperature monitoring (Figure 8b) confirmed substantial heating, reaching 62.7 °C at 15 min in the presence of MXene nanosheets, compared to only 31.1 °C in the MXene-free control (Figure 8c). SEM imaging confirmed bacterial cell damage following NIR exposure, with *S. aureus* exhibiting pronounced surface cracking and extensive membrane indentations. Similarly, *E. coli* cells displayed multiple surface deformations accompanied by apparent leakage of intracellular contents, indicating structural disruption induced by photothermal treatment (Supporting Information, Figure S13). However, bacterial regrowth was observed 24 h post-treatment in the 5 min exposure group, as confirmed by spectrophotometric analysis. These findings indicate that MXene-assisted PTT exhibits strong immediate antibacterial effects. Still, its long-term efficacy may be limited by bacterial resilience, environmental conditions, or incomplete inactivation of residual bacteria. This suggests that repeated or prolonged PTT exposure may be necessary to achieve sustained antibacterial outcomes.

An alternative strategy to enhance the antibacterial efficacy of PTT while minimizing off-target damage is to apply targeted PTT ablation, which allows for selective bacterial elimination and reduces harm to surrounding tissues. In previous work, we developed a MXene–polydopamine–antibody (MXene-PDA-Ab) complex for selective melanoma cell ablation, demonstrating high specificity and effectiveness.^33^ In the present study, we extended this concept to antibacterial therapy by functionalizing the MXene-PDA complex with ABIN3027591 antibodies, which specifically target *E. coli* surface antigens. *S. aureus* was used as a control, as it lacks the corresponding antigen and thus does not bind the antibody-functionalized complex.

Following 2 h of coincubation with either *E. coli* or *S. aureus*, unbound MXene-PDA-Ab complexes were washed away, and the samples were subjected to NIR laser irradiation. As shown in Figure 9a, no bacterial growth was detected in the ABIN3027591-positive *E. coli* group via both live/dead staining and agar plating, indicating effective and selective eradication of the targeted bacteria. In contrast, *S. aureus* (which lacks the ABIN3027591 antigen) continued to grow (Figure 9a), and no evidence of bacterial death was observed following PTT exposure (Figure 9b). These findings confirm the feasibility of antigen-targeted MXene-assisted PTT as a precise and efficient method for selective bacterial ablation.

**Figure 9:**
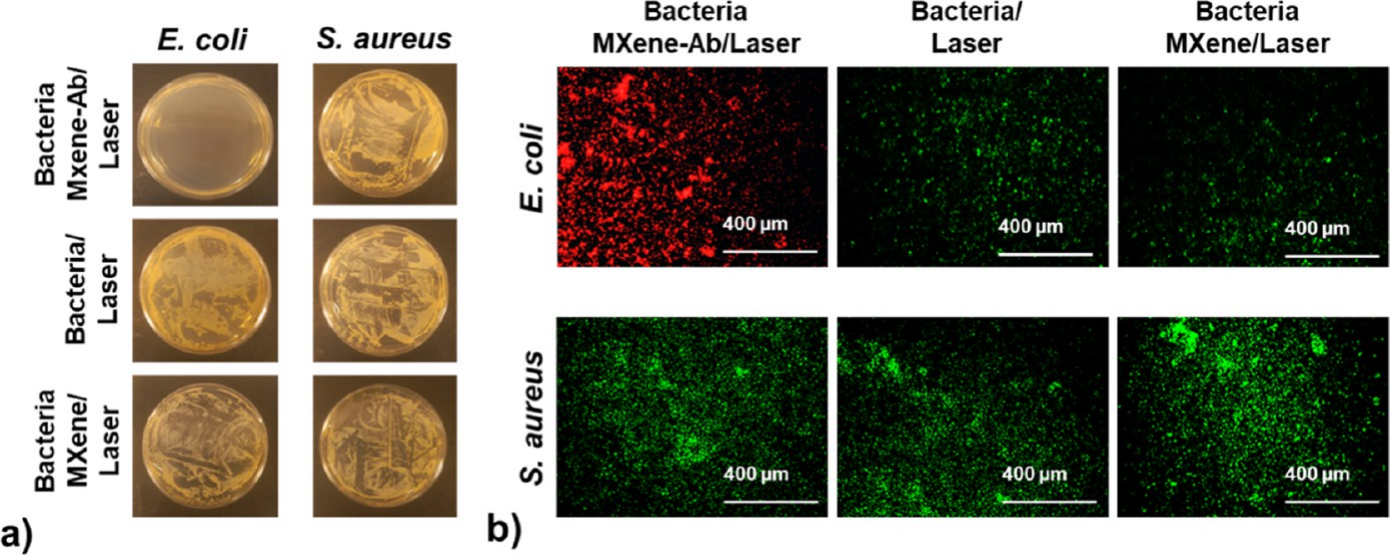
Representative images of *S. aureus* and *E. coli* growth on Mueller–Hinton agar (MHA) plates after 24 h of incubation (a), and corresponding live/dead fluorescence images (b) obtained after Ti_3_C_2_T_*x*_ MXene-based laser treatments, demonstrating photothermal therapy PTT-induced bacterial ablation.

Both experiments demonstrate the potential of MXene-assisted photothermal therapy (PTT) as an effective antibacterial strategy. Together, these findings confirm that both general and targeted PTT using MXenes can achieve effective bacterial killing, but targeting strategies offer a path to precision antimicrobial interventions with reduced risk to nontarget cells or tissues. To validate the effectiveness of the antibacterial PTT strategy, we conducted an in vivo animal trial to assess its potential for clinical translation.

### 3.6 In Vivo Validation of MXene-Based PPT

Before the in vivo experiment, we provide a comprehensive assessment of Ti_3_C_2_T_*x*_ MXene-based NIR photothermal performance to select appropriate PTT regimens, safety of selected regimens toward cell culture, and antibacterial effectiveness with *E. coli*, *S. aureus*, and *P. aeruginosa* (including their mixture). We employed a 755 nm EVOLINE-1 pulsed laser (8 ms pulse duration, 10 J/cm^2^ fluence, 1.0 Hz frequency) with various exposure durations and interval settings. Based on the recorded temperature dynamics (see Supporting Information, Figure S14), four irradiation time regimens were selected for in vitro evaluation: R1 (10 + 10 + 10 s), R2 (20 s), R3 (15 s), and R4 (5 + 5 + 5 s). None of the tested regimens exhibited cytotoxic effects; on the contrary, all demonstrated a mild stimulatory effect on cell proliferation (Figure 10a). Previous studies have shown that low-dose near-infrared (NIR) irradiation can enhance cellular metabolism and proliferation. This is primarily mediated by the activation of redox-sensitive signaling pathways, including Nrf2, NF-κB, and ERK,^49^ as well as the stimulation of mitochondrial respiration through the interaction with endogenous porphyrins or cytochrome complexes.^50^ Morphological assessment further confirmed the safety of NIR laser exposure under the selected conditions, with no signs of cellular damage or abnormal morphology observed (Figure 10b).

**Figure 10:**
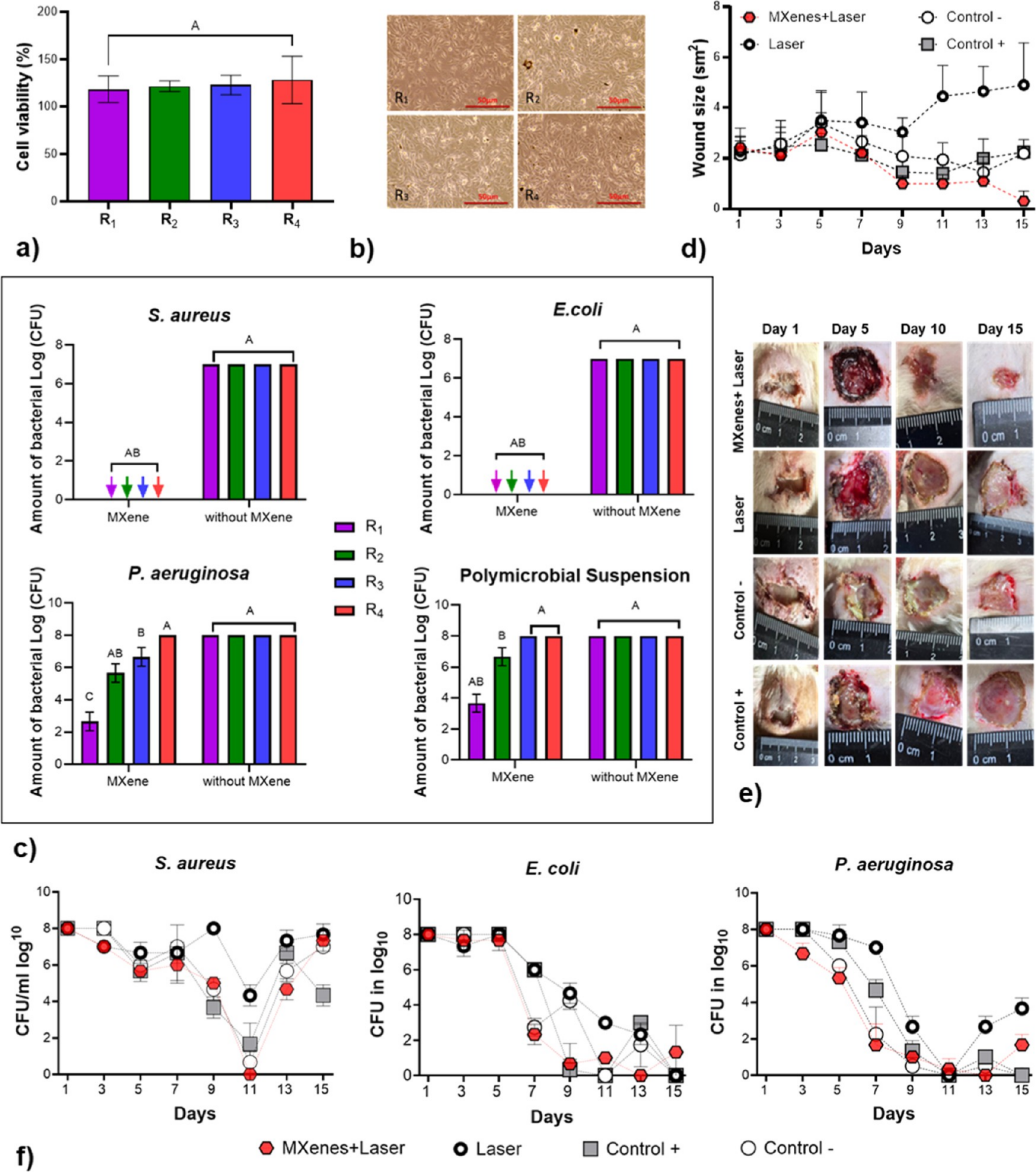
Results of cell viability (a) with cell morphology, (b) after pulsed NIR laser exposure in different regimens: R1 (10 + 10 + 10 s), R2 (20 s continuous), R3 (15 s continuous), and R4 (5 + 5 + 5 s), with data of in vitro bacteria survival after irradiation in the same regimens (c). Dynamics of wound area changes across experimental groups over 15 days (d), macroscopic images of wound defects at different time intervals of the experiment (e), and dynamics of bacterial survival in vivo after NIR irradiation (regimen R1) (f). Statistical significance was determined using the compact letter display (CLD). Groups labeled with the same letters are not significantly different, while groups labeled with different letters show statistically significant differences (*p* ≤ 0.05).

During the 24 h experimental period, the isolated bacterial strains (*E. coli*, *S. aureus*, and *P. aeruginosa*) reached concentrations of approximately 10^8^ CFU. Exposure to NIR irradiation alone (without MXene) did not significantly inhibit bacterial growth in any of the tested regimens. However, the incorporation of MXenes into the bacterial culture media resulted in a complete elimination of *E. coli* and *S. aureus* across all irradiation regimens (Figure 10c). For *P. aeruginosa*, a significant reduction in viability was observed only under the R1 regimen. A similar trend was noted for the mixed bacterial culture, which exhibited sensitivity exclusively under the R1 irradiation condition. Based on biocompatibility tests and bacteria sensitivity results, the R1 (10 + 10 + 10 s) regimen was selected for the in vivo trial.

The R1 regimen (10 + 10 + 10 s) was selected for the in vivo trial as it demonstrated the highest efficacy and safety profile. NIR irradiation was performed once daily over 3 days to ensure effective bacterial ablation within the wound area. Temperature dynamics at the wound site were monitored before and after laser irradiation (Supporting Information, Figure 15a). Baseline temperatures in both the laser-only and MXene + laser groups ranged from approximately 30 to 35 °C. Following NIR laser exposure, all groups exhibited a rapid temperature increase, reaching peak values between 65 and 70 °C. Notably, the MXene + laser group demonstrated slightly higher postirradiation temperatures (∼68–70 °C) compared to the laser-only group (∼65–68 °C), suggesting enhanced photothermal conversion efficiency in the presence of MXenes (Supporting Information, Figure 15 b). The observed temperature elevation in the laser-only group may be attributed to the absorption of NIR light by the dark-colored wound scab, which could enhance local photothermal effects through increased light absorption.

The combined treatment with MXenes and laser irradiation resulted in the most effective and statistically significant acceleration of wound healing (Figure 10d,e). A notable reduction in wound area was observed as early as day 3, with continued improvement on days 7 and 15. In comparison, laser treatment alone and Betadine application also promoted wound contraction, though their effectiveness was lower than that of the combined therapy.

MXene-assisted photothermal therapy also resulted in a marked reduction of bacterial load in purulent wounds (Figure 10f). In the MXene + laser group, the numbers of *S. aureus*, *E. coli*, and *P. aeruginosa* decreased sharply by day 5 and continued to decline through days 10 and 15, reaching near-complete suppression of bacterial growth. Laser treatment alone and Betadine application demonstrated moderate antibacterial effects, with reduced bacterial counts compared to control, but less effective compared to combined treatment. In the rat model of purulent wound infection, a reduction in wound area was observed by day 10. However, all inoculated pathogens, particularly *S. aureus*, remained detectable. This discrepancy between visible wound contraction and incomplete microbial clearance has been reported in polymicrobial infections, where interactions between *S. aureus* and *P. aeruginosa* support persistent colonization.^51^ *S. aureus* frequently survives within biofilms and benefits from microenvironmental changes during the healing process.^52^ Moreover, the host microbiome can influence infection dynamics by interacting with invading pathogens, thereby modulating biofilm formation and persistence. This highlights the importance of integrating clinical, histological, culture, and microbiome analyses to comprehensively interpret healing dynamics. Overall, these findings emphasize the superior efficacy of the combined MXene-laser therapy compared to other treatment approaches.

## 4 Discussion

In the present study, MXene nanosheets did not demonstrate significant antibacterial activity, a result that contrasts with numerous earlier reports. Previous studies have reported measurable bactericidal effects of Ti_3_C_2_T_*x*_ MXene against both Gram-positive and Gram-negative bacteria. For instance, Rasool et al. observed that fresh Ti_3_C_2_T_*x*_ MXene membranes could inhibit ∼67–73% of *E. coli* and *B. subtilis* growth, and that aged (oxidized) MXene membranes achieved >99% inhibition.^7^ Such data led to an expectation of intrinsic MXene antibacterial properties. However, our findings suggest that pristine, high-quality Ti-, V-, and Nb-based MXenes are largely inert as antibacterial agents under standard assay conditions. Also, there is considerable inconsistency in the literature regarding the relative sensitivity of Gram-positive and Gram-negative bacteria to Ti_3_C_2_T_*x*_ MXene. Some studies report that Gram-negative *E. coli* is more susceptible than Gram-positive *B. subtilis*, attributing this to the thinner peptidoglycan layer in *E. coli*.^5^ Conversely, other reports suggest enhanced sensitivity of *B. subtilis*, likely due to the absence of an outer membrane and the presence of a more negatively charged cell surface at physiological pH, which could strengthen electrostatic interactions with Ti_3_C_2_T_*x*_. These contradictory findings are partly explained by differences in MXene formulations - some studies employed micrometer-thick Ti_3_C_2_T_*x*_ membranes, while others used delaminated nanosheets in suspension. In our study, Gram-positive *S. aureus* exhibited slightly higher sensitivity to Ti_3_C_2_T_*x*_ MXene than Gram-negative *E. coli*, partially aligning with observations from studies using suspended nanosheets. This discrepancy urges a critical examination of material differences and experimental factors that could explain why prior literature reported stronger effects.

One key factor is the composition and purity of the MXene nanosheets, which were not well controlled at the early stages of MXene research. The MXene flakes in this study were of high purity, as confirmed by extensive characterization (Figures 1 and S2). Our data showed no peaks for TiO_2_ or other oxides, and surface chemistry characterization indicated predominantly –O/–OH terminations with only small –F content in Ti-based MXenes, reflecting minimal remnants of etchants. In contrast, earlier studies may have inadvertently tested MXenes with partial oxidation or residual etchant species, which can artificially boost apparent antibacterial activity. Rasool et al. noted that surface oxidation of Ti_3_C_2_T_*x*_ (upon aging) led to the formation of anatase TiO_2_ nanocrystals on MXene surfaces.^7^ Anatase TiO_2_ is a known catalyst for ROS generation; even under ambient conditions, it can produce radicals that damage bacterial cells.^53^ Thus, oxidized MXenes likely kill bacteria via ROS-mediated oxidative stress, an effect not intrinsic to the MXene itself but to its oxide byproducts. Because our MXenes were stored and handled under inert conditions to prevent oxidation, this ROS burst mechanism was largely absent. This explains why we did not observe the strong antibacterial effect that an “aged” or oxidized MXene might exhibit. Likewise, MXenes synthesized by selective etching in HF-containing solutions often retain a high concentration of fluorine surface terminations that can hydrolyze, forming HF, or contaminants (e.g., intercalated aluminum fluoride or residual HF). If not thoroughly washed to pH 6–7, such residues can leach out and induce cell death, providing antibacterial properties, but also toxicity to MXene.^10^ Even if all HF and AlF_3_ had been removed, hydrolysis of Ti–F bonds of MXenes etched in concentrated HF may generate HF in situ. In our work, however, rigorous purification removed most of the HF-derived species, and the use of a low concentration of HF minimized the fluoride terminations. The lack of antibacterial activity underscores that pure MXene carbides are relatively biocompatible.

Differences in synthesis and morphology may offer another explanation for the divergent results. Antibacterial action of 2D materials often depends on structural features like sheet size, thickness, and edge sharpness. A recent study demonstrated that MXene prepared by mechanical exfoliation (instead of conventional chemical etching) yielded flakes with abundant irregular sharp edges, acting as “nano-knives” that physically disrupt bacterial membranes.^54^ These edge-rich MXene sheets showed superior antibacterial activity compared to smoother sheets from standard etching. In our study, MXene nanosheets were synthesized via wet chemical etching, producing relatively large, flat, primarily single-layer sheets with fewer jagged edges. Such high-quality sheets may cause less damage to bacterial membranes than the defect-rich or deliberately crumpled MXenes.

Another important issue worth discussing is the apparent mismatch between the reported biocompatibility and antibacterial activity of MXenes in previous studies. In principle, biocompatibility (minimal toxicity to mammalian cells) and antibacterial efficacy are not theoretically incompatible; however, nanomaterials that kill bacteria via nonspecific mechanisms typically exhibit some degree of toxicity toward mammalian cells, unless specifically engineered otherwise. A large number of publications describe simultaneously high biocompatibility and strong antibacterial effects of various MXene formulations. However, this raises a mechanistic contradiction: it is well established that MXenes do not possess specific antibacterial mechanisms such as enzyme inhibition or interference with metabolic pathways.^43,55^ Rather, their antibacterial activity is typically attributed to nonspecific mechanisms, namely, ROS-mediated oxidative stress and mechanical membrane disruption (the so-called “nano-knife” effect). These processes indiscriminately damage fundamental biomolecules and cell structures (e.g., membranes, proteins, lipids, DNA), and ROS in particular have no intrinsic ability to distinguish bacterial cells from mammalian cells.^56^ As a result, potent antibacterial effects achieved through such nonselective pathways often coincide with cytotoxic effects on host cells. Indeed, many conventional nanoantibacterials (for example, highly cationic nanoparticles) display excellent bactericidal activity but also cause significant mammalian cell injury (e.g., membrane lysis or oxidative damage), making their safe biomedical application challenging.^57^ Achieving truly selective antibacterial action usually requires incorporating specific targeting strategies or surface modifications to mitigate nonspecific interactions.^58^ In the absence of a selective bacterial target (such as a unique enzyme or metabolic pathway), it is difficult for a material to exert strong bactericidal effects without also harming mammalian cells.^59^ Unless MXenes are specially functionalized to confer selectivity, their broad mechanisms of action imply that strong antibacterial effects will likely be accompanied by at least some degree of mammalian cytotoxicity—a reality that reinforces the validity of this apparent contradiction.

In our study, Ti-based MXene nanosheets exhibited excellent biocompatibility. At the same time, V_2_CT_*x*_ and Nb_2_CT_*x*_ demonstrated moderate cytotoxicity, likely due to their rapid oxidation in biological media (Figure 2). This cytotoxicity pattern correlated closely with their limited antibacterial activity, further supporting the idea that previously reported antibacterial effects may have arisen from oxidative degradation products rather than intrinsic MXene properties. Moreover, our calculations suggest (Supporting Information Figure S3, Table S1) that even at relatively low concentrations (100 μg/mL), a single *E. coli* cell may encounter approximately 17,000 MXene flakes with a 1300 nm lateral size (and more than 100,000 if the lateral size is 500 nm) during coincubation. Given that mammalian cells are 10–20 times larger, they would be expected to encounter many more MXene flakes under the same conditions—more than enough to cause mechanical injury if the “nanoknife” mechanism were broadly active. Here, it is important to note that the apparent discrepancy between the calculated numbers and the SEM/TEM observations regarding the number of MXene flakes could be attributed to the sample preparation procedure. During SEM/TEM sample processing, multiple washing steps are performed, which remove unbound flakes, leaving only those that are associated with the cells or bacteria to be visualized.

Another important factor influencing MXene-bacteria interactions is the formation of a protein corona in biological environments, which significantly alters the material’s surface properties. When introduced into physiological fluids, MXenes rapidly adsorb proteins and other biomolecules, forming a dynamic corona that often reduces surface hydrophilicity and shields reactive functional groups and sharp edges. This passivation can weaken direct physical and chemical interactions with bacterial cells that are essential for membrane disruption and oxidative damage. Additionally, pristine MXenes possess a net negative surface charge, resulting in electrostatic repulsion with negatively charged bacterial membranes. The formation of a protein corona can modulate this repulsion by partially neutralizing the surface charge or altering its distribution, depending on the specific proteins involved. In some cases, this may reduce electrostatic barriers and facilitate bacterial contact; however, a dense or negatively charged corona may instead enhance steric and electrostatic hindrance. Consequently, the presence of a protein corona is more likely to diminish the antibacterial efficacy of MXenes by limiting their ability to adhere to and disrupt bacterial membranes. Importantly, while the role of protein coronas in modifying surface charge, colloidal stability, and cellular uptake has been investigated,^60^ most existing studies focus on mammalian cells or general physicochemical behavior, not on interactions with bacteria.

Yet, our experimental results and prior studies, including our own, show no evidence of membrane damage or metabolic disruption in mammalian cells following MXene exposure.^61–63^ Indeed, MXene flakes smaller than 1 μm have been shown to internalize readily into mammalian cells without inducing cytotoxicity or interfering with cell metabolism.^23^ These findings suggest that well-prepared, oxidation-free MXene nanosheets lacking toxic residues tend to exhibit low or negligible antibacterial activity, while retaining high biocompatibility. The absence of any “nano-knife” membrane-cutting effect in our experiments can be explained by MXene’s surface chemistry. Ti_3_C_2_T_*x*_ MXenes are highly hydrophilic due to their terminating –OH/-O groups, which create a substantial energy barrier to inserting into lipid bilayers. In other words, strong repulsive interactions between the MXene’s polar surface and the hydrophobic interior of bacterial membranes prevent the flakes from puncturing the cell envelope. Consistent with simulation studies, we note that MXene flakes would require an order-of-magnitude higher force (∼10 nN) to penetrate a membrane compared to more hydrophobic 2D materials like graphene (∼2 nN).^64^ This suggests that MXenes are far more likely to remain attached to the external membrane surface rather than slicing through it, especially under typical biological conditions. Indeed, Ti_3_C_2_T_*x*_ nanosheets prefer to lie flat along the bacterial cell wall, adhering via surface attractions, instead of behaving as nanoscalpels. Such an orientation minimizes direct mechanical disruption to the membrane, which explains why no physical cell membrane rupture was observed in our study. Rather than contradicting previous reports, our data provide a more refined understanding: the intrinsic antibacterial activity of MXenes is limited, and strong effects reported in earlier studies were likely influenced by material degradation, oxidation byproducts, impurities (etching products), or highly irregular nanosheet morphologies that enhance physical interaction with bacterial membranes. It is also possible that incomplete removal of LiF or other salts used for delaminating MXenes contributed to the observed effects. Finally, etching for a prolonged time, in concentrated HF or at elevated temperature, may create point defects and pinholes^65^ that are more reactive than the basal plane of MXene and may affect antibacterial properties. All these factors need a separate systematic study to determine which of them may lead to antibacterial properties and/or cytotoxicity of MXenes.

It is also important that our findings highlight the challenges of evaluating MXenes’ antimicrobials with traditional assays. Standard methods like disk diffusion and broth microdilution were developed for soluble antibiotics and may overlook or exaggerate effects when applied to 2D nanomaterials. In a disk diffusion test, a lack of an inhibition zone around a sample-impregnated disk is usually interpreted as no antibacterial activity. However, nanoparticles and nanosheets do not readily diffuse through agar, so the absence of a clear zone of inhibition does not necessarily mean the material is ineffective—it may simply never reach the bacteria in the agar medium. Conversely, any zone that does appear in such tests often results from diffusible byproducts (e.g., metal ions, soluble radicals) rather than the nanoparticles themselves.^66^ In the case of MXene, this means a disk diffusion assay would primarily reflect whether the MXene releases any antibacterial species (like HF, metal ions or peroxide); a pristine Ti_3_C_2_T_*x*_ that relies on direct contact killing would register little to no zone, as was observed in our experiments (Supporting Information, Figure S7). Thus, agar diffusion inherently underestimates contact-dependent antibacterial effects and can mislead researchers about a nanomaterial’s true efficacy.

Broth-based MIC assays, on the other hand, allow direct contact between nanomaterials and bacteria in liquid suspension. While this overcomes the physical delivery issue, it introduces new artifacts. Aggregation of 2D materials in rich media can significantly influence outcomes. MXene sheets tend to aggregate or sediment in ionic solutions; aggregated MXenes might entrap bacteria in clumps, causing an apparent reduction in colony count without actually killing them, or conversely, they might settle out and reduce effective exposure. Moreover, optical interference is a concern as MXenes are highly light-absorbing and can scatter light. In a turbidity-based microdilution readout, dense MXene suspensions and even lysed cell debris can raise the apparent optical density, confusing viability measurements.^67^ Our study took precautions (including using plating methods and metabolic dyes in addition to OD measurements) to ensure that any growth inhibition was due to genuine bactericidal/bacteriostatic effects rather than such interference. Some prior studies may have overestimated antibacterial potency due to transient phenomena. For example, the initial mixing of MXenes into an oxygenated broth could trigger a short-lived ROS burst (as fresh MXene surfaces oxidize) that damages bacteria at the early stage. If measurements are taken at that point, one might conclude a strong antibacterial effect, even though the surviving bacteria could recover once the ROS are depleted. Similarly, if MXene-bacteria aggregates form, they may settle out of suspension, leading to lower observable colony counts in the supernatant without thorough mixing. By recognizing these limitations, our work underscores that traditional antimicrobial assays must be carefully adapted for nanomaterials. Consistent with recent critiques, a combination of multiple testing methods is recommended to validate the antimicrobial performance of nanomaterials.^66^

In light of the discussion above, a key question remains: Do MXenes hold genuine promise as antibacterial agents? While pristine MXenes showed little intrinsic antibacterial action at concentrations that were above the onset of toxicity, our study demonstrates that they can be extremely potent when used as agents in photothermal therapy. MXenes (especially Ti_3_C_2_T_*x*_) possess strong broadband absorption and efficient conversion of light to heat.^68^ In fact, Ti_3_C_2_T_*x*_ nanosheets exhibit photothermal conversion efficiencies around 30–50%, outperforming traditional photothermal materials, such as gold nanorods.^4^ In this research, we leveraged these properties where MXene-based PTT achieved rapid and targeted bacterial ablation (Figures 8 and 9). Under NIR laser irradiation, photothermal heating is the predominant mechanism by which MXenes exert bactericidal effects. Prior studies have shown that Ti_3_C_2_T_*x*_ MXene combined with 808 nm light can rapidly kill bacteria via localized thermal damage, rather than through chemical reactive oxygen species.^65^ In our system, the MXene flakes, once bound to the bacterial surface, act as nanoheaters that convert light energy to heat with high efficiency. Importantly, the interaction at the MXene–bacteria interface (hydrogen bonding and electrostatic attraction between MXene’s surface and the cell envelope) enables ultrafast heat transfer into the membrane.^70^ Femtosecond spectroscopy measurements have revealed that ∼80% of the photoexcited energy in Ti_3_C_2_T_*x*_ dissipates into the surrounding water (or biological medium) within a few picoseconds via these interfacial pathways. This means that when MXene flakes are illuminated, they promptly channel thermal energy into nearby bacterial membranes, producing a highly localized “hot spot”. The result is a rapid rise in temperature at the cell surface, causing irreversible membrane disruption and cell lysis in minutes.^69^ Notably, this photothermal ablation occurs with minimal contribution from ROS generation or other chemical means, as the process is fundamentally a physical heat-induced membrane damage.

This finding is in line with Rosenkranz’s report that MXenes combined with NIR laser exposure cause irreversible bacterial cell damage (with treated cells reduced to debris), whereas bacteria exposed to MXene alone can eventually regrow.^19^ In our experiments, upon NIR irradiation, we observed complete eradication of the bacterial population in vitro, with no regrowth in subsequent culture, confirming that PTT converted MXenes from passive nanomaterials into highly active antibacterial agents.

Crucially, our PTT approach was designed with selectivity and biocompatibility in mind. By conjugating MXene flakes with specific antibodies, we endowed the nanomaterial with the ability to selectively bind to target bacteria. This antibody-functionalized MXene homed in on the bacterial cells of interest (e.g., a particular pathogen in a mixed sample), decorating their surfaces. NIR laser exposure then produced intense local heating (>50 °C) at those antibody-tagged bacteria, leading to their destruction, while unbound MXenes in solution and nearby nontargeted cells experienced much milder heating. The result is a form of targeted photothermal ablation, where the pathogen is selectively eliminated with minimal collateral damage to surrounding beneficial microbes or host tissues. Selectivity is a critical advantage because indiscriminate heating could harm host cells; by focusing the thermal effect through molecular recognition (antibody–antigen binding), we enhance safety. Indeed, throughout our PTT experiments, the MXene-antibody bioconjugates showed good biocompatibility with mammalian cells (no significant toxicity in the absence of laser), and the heat generation was confined both spatially and temporally—only upon laser activation and primarily at the sites of bound bacteria. The artificial protein corona formed on MXene surfaces significantly enhances colloidal stability by preventing aggregation in biological environments. The effect of the protein corona depends on protein concentration: at lower concentrations, it may destabilize MXene dispersions and promote aggregation, while at higher concentrations, a dense protein layer forms on the MXene surface, generating steric repulsion that improves dispersion stability.^71^ Although the corona’s composition can enhance MXene dispersion, it may also reduce direct antimicrobial activity by limiting physicochemical interactions. This highlights the need for targeted strategies to enhance selectivity and reduce off-target effects. Antibody-functionalized MXene complexes, such as MXene-PDA-Ab, enable selective binding to specific bacteria like *E. coli*, compensating for the protein corona’s dampening effects. Under near-infrared irradiation, this approach allows precise bacterial elimination while minimizing harm to surrounding tissues and microbes. Thus, balancing protein corona effects with antibody-mediated targeting is crucial for optimizing MXene antibacterial therapies. Such on-demand, controllable bactericidal action is especially promising against antibiotic-resistant bacteria, as it operates via a physical mechanism (thermal damage) to which bacteria cannot easily develop resistance. Furthermore, unlike antibiotics, photothermal killing does not rely on bacterial metabolism or replication, so it remains effective against planktonic or biofilm-embedded bacteria when targeted. Crucially, we validated the in vivo applicability of MXene-assisted photothermal antibacterial therapy, highlighting its potential for future clinical translation and the development of next-generation antibacterial approaches.

The answer to the key question is positive—MXene nanosheets exhibit great promise as antibacterial agents, even though pristine MXenes alone are not strongly antibacterial in the conventional sense. However, their unique combination of properties, tunable surface chemistry, excellent photothermal conversion efficiency, high specific surface area, and ease of biofunctionalization can be harnessed in more sophisticated and clinically relevant ways to combat microbes. The successful antibody-targeted PTT demonstrated a conceptual shift from viewing MXenes as simple “nano-antibiotics” toward using them as multifunctional nanoplatforms that integrate biorecognition for selectivity, nanothermal effects for lethality, and intrinsic biocompatibility for safety, in line with the demands of modern nanomedicine. Going forward, this platform concept can be extended by coupling MXenes not only with antibodies but also with other targeting ligands and therapeutic components (e.g., antimicrobial peptides, cytokines, or growth factors that support tissue regeneration), enabling combined antimicrobial and pro-healing functions in infected wounds or implant-associated infections. At the same time, further fundamental studies on MXene–cell and MXene–bacteria interactions, optimization of surface terminations, and systematic evaluation of long-term safety will be essential to refine selectivity and define safe operating windows for in vivo use. Thus, our findings encourage future work that develops MXene-based photothermal and multimodal antimicrobial systems for applications such as smart wound dressings, implant coatings, and localized infection control. In this context, the lack of strong direct killing at low concentrations should be regarded not as a limitation but as an opportunity to deploy MXenes as externally triggered, precisely controllable antibacterial platforms rather than as conventional, continuously active biocides.

## 5 Conclusions

Contrary to several prior reports suggesting strong intrinsic antibacterial activity of MXene nanosheets, our systematic investigation of high-quality pristine Ti-, V-, and Nb-based MXenes using a suite of in vitro and in vivo models demonstrates that high-quality stoichiometric, minimally oxidized MXenes exhibit negligible antimicrobial effects under standard conditions. We found no support for either of the two frequently proposed antibacterial mechanisms—reactive oxygen species generation or membrane disruption via the “nano-knife” effect, at biologically relevant, subtoxic concentrations. Thus, MXene nanosheets under study do not possess intrinsic antibacterial properties. Antibacterial effects observed in prior studies, when MXene research was just emerging, are likely explained by defects, the presence of etching residues such as HF or AlF_3_, fluorine-rich surface terminations, and sample oxidation.

Our work emphasizes that pristine MXene nanosheets, when free from oxidation byproducts and etching residues, are highly biocompatible but possess only limited antibacterial potential. Nonetheless, we demonstrate that the unique photothermal properties of MXenes can be harnessed for highly effective bacterial ablation. MXene-assisted photothermal therapy, especially when combined with antibody-based targeting, resulted in rapid and selective bacterial eradication in vitro and was successfully translated to an in vivo wound model. This strategy enables spatially confined, on-demand antibacterial action with minimal damage to host tissues, addressing one of the major limitations of traditional antibiotic and nanomaterial-based approaches.

Taken together, our results redefine the role of MXenes in antimicrobial applications, not as direct bactericides, but as versatile, biocompatible platforms that can be activated for targeted photothermal disinfection. This work lays the foundation for the development of next-generation MXene-based antimicrobial strategies, particularly in the context of multidrug-resistant infections and localized wound management. Future studies should focus on optimizing MXene nanosheet formulations for clinical use, including surface functionalization, delivery vehicles, and real-time imaging capabilities.

## Supplementary Material



## Data Availability

Data will be made available on request.

## Notes

Figs. S1–S4 show experimental setup for Ti_3_C_2_T_*x*_ MXene-bacteria co-cultivation, samples preparation for SEM observation, calculation of MXene-bacterial interaction, and MXene-assisted photothermal ablation protocol; Figs. S5–S6 show results of DLS measurements and TEM images of Ti_3_C_2_T_*x*_, Nb_2_CT_*x*_, V_2_CT_*x*_, and Ti_3_CNT_*x*_ MXenes; Figs. S7–S10 show additional data from antibacterial experiments with MXenes; Figs. S11–S12 show SEM and TEM images (with EDX mapping) of MXene-bacteria interactions; Fig. S13 shows SEM images of the S. aureus and E. coli after Ti_3_C_2_T_*x*_ MXene-based photothermal therapy; Figs. S14–S15 show selection of laser regimens with the representative temperature growth after the NIR irradiation and dynamics of in-vivo wound temperature changes with the thermal images; Table 1 shows calculated parameters of Ti_3_C_2_T_*x*_ MXene water dispersions (PDF)
