# Supplementary material for: Critical Assessment of Intrinsic Antibacterial Properties and Photothermal Therapy Potential of MXene Nanosheets
Source: ACS Appl Nano Mater. 2026 Jan 13;9(4):1925–48. doi: 10.1021/acsanm.5c04961 (PMC12865921; doi:10.1021/acsanm.5c04961)
Supplement: Supplementary file 1 [file an5c04961_si_001.pdf]

Supporting Information

Critical Assessment of Intrinsic Antibacterial Properties and Photothermal Therapy Potential of MXene Nanosheets

Viktoriia Korniienko[1,2], Oleksiy Gogotsi[2,3,4], Yuliia Varava[2], Baiba Zandersone[1], Volodymyr Deineka[1,2], Yevheniia Husak[2,5], Kateryna Diedkova[1,2], Oleksandr Solodovnyk[2,6], Vjacheslav Kukurika[2], Serhii Dukhnovskiy[3], Roman Moskalenko[2], Ivan Baginskiy[3], Oksana Petrichenko[7], Oksana Sulaieva[8], Olena Haidamak[8], Pavlo Shubin[1], Veronika Zahorodna[3], Błażej Anastaziak[9], Emerson Coy[9], Igor Iatsunskyi[9], Yury Gogotsi[2,10*], Maksym Pogorielov[1,2*]

[1]Institute of Atomic Physics and Spectroscopy, Faculty of Science and Technology, University of Latvia, Jelgavas 3, LV-1004 Riga, Latvia
[2]Biomedical Research Centre, Sumy State University, 40007 Sumy, Ukraine
[3]Materials Research Centre, 3 Krzhizhanovskogo Str., 03142 Kyiv, Ukraine
[4]NanoCarbonTech, Rubiez 46, 61-612 Poznan, Poland
[5]Faculty of Chemistry, Silesian University of Technology, Strzody 9, 44-100 Gliwice, Poland
[6]Werba Medical, 40035 Sumy, Ukraine
[7]Department of Physics, Faculty of Science and Technology, University of Latvia, Jelgavas 3, LV-1004 Riga, Latvia
[8]Medical Laboratory CSD, Vasylkivska 45, 02000 Kyiv, Ukraine
[9]NanoBioMedical Centre, Adam Mickiewicz University, 3, Wszechnicy Piastowskiej Str., 61-614 Poznan, Poland
[10]A. J. Drexel Nanomaterials Institute, and Department of Materials Science and Engineering, Drexel University, Philadelphia, Pennsylvania 19104, USA

*Corresponding authors: Yugy Gogotsi (yg36@drexel.edu), Maksym Pogorielov (maksym.pogorielov@lu.lv)

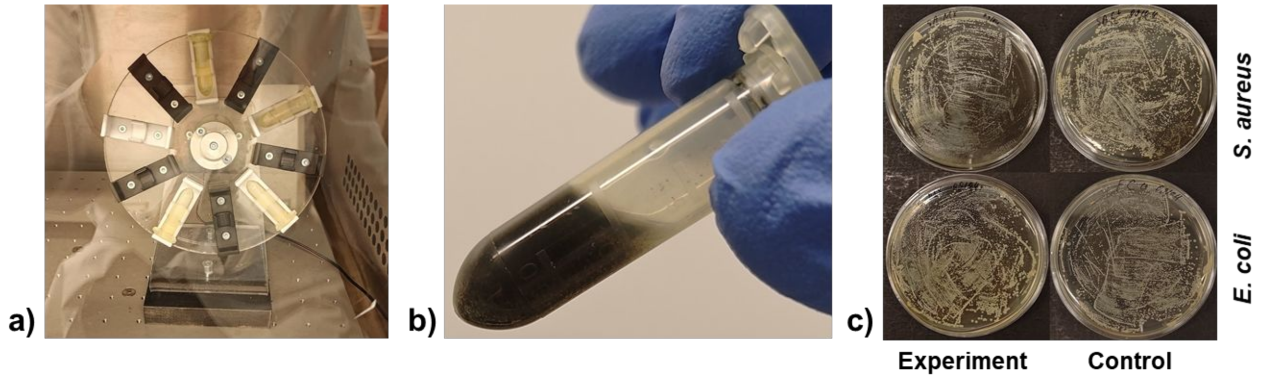

Figure S1. Rotational Incubation Setup for $Ti_3C_2T_x$ MXene-bacteria co-cultivation: (a) Custom-built rotator to maintain $Ti_3C_2T_x$ MXene suspension; (b) Sedimentation of Ti_3C_2 MXene observed in static conditions with (c) - representative images of *S. aureus* and *E. coli* growth on MHA plates after 24-hour co-incubation with Ti_3C_2 MXene (400 µg/mL) under rotational conditions: experimental group (co-cultivation of MXenes with bacteria) and the control group (bacteria without MXenes).

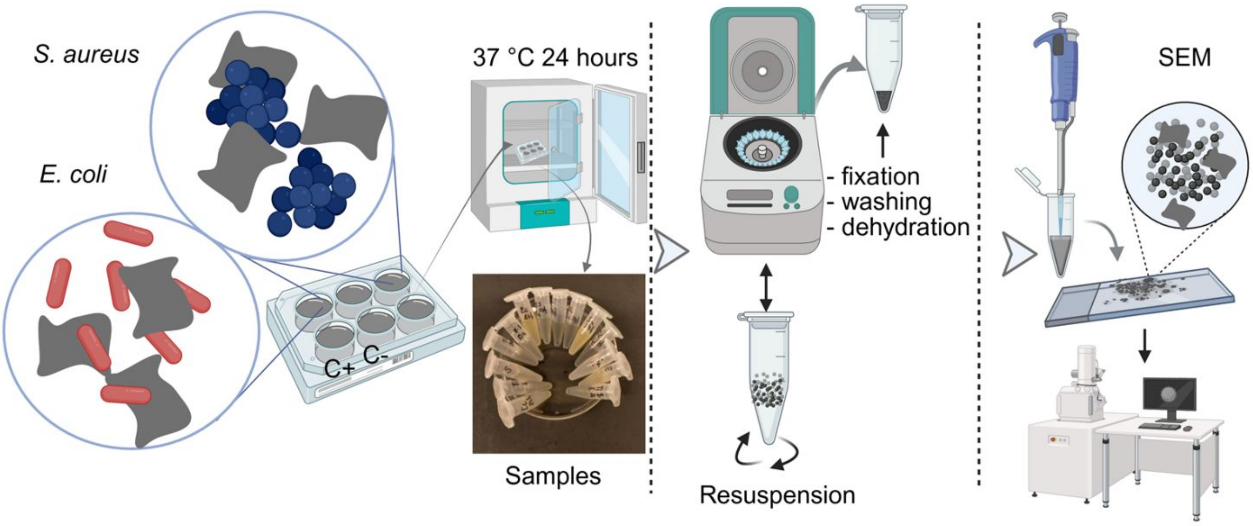

Figure S2. Schematic representation of samples preparation for SEM observation.

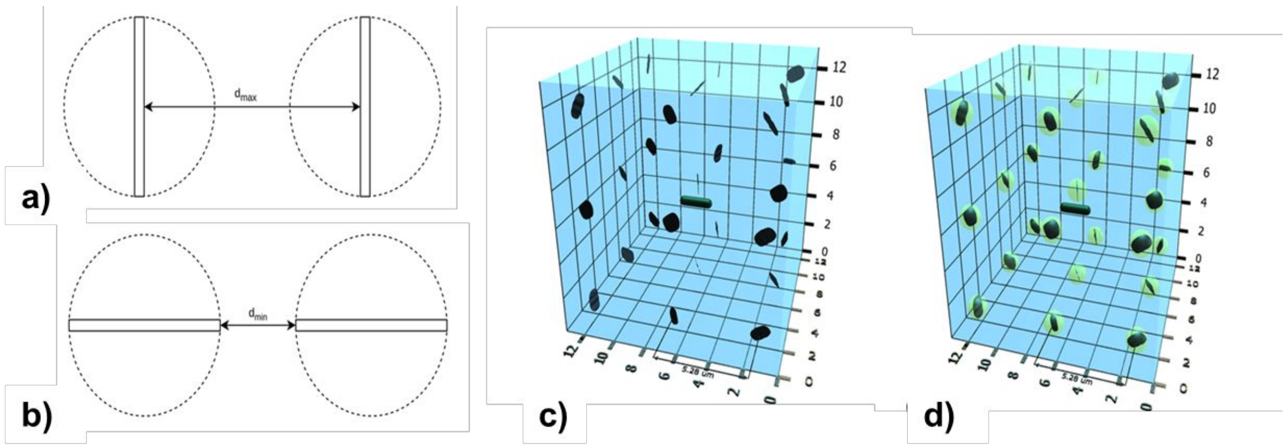

Figure S3. (a) Two types of relative orientation of Ti_3C_2 MXene flakes and (b) parallel orientation that corresponds to maximum distance d_{max} between flakes and (b) in-plane orientation that corresponds to

minimum distance between flakes d_{min}. (c) MXene flakes surrounding bacteria; (d) MXene flakes surrounding bacteria with consideration of volume each flake can take due to variation in orientation.

Table S1. Calculated parameters of $Ti_3C_2T_x$ MXene water dispersions at 100 μg/mL for two flake sizes

Parameter	1300 X 900 nm flakes	500 X 350 nm flakes
**The number of MXene flakes per 1 mL, N**	$1.77 \cdot 10^{10}$	$1.18 \cdot 10^{11}$
**Maximum distance between flakes, d_{max} (μm)**	3.84	2.04
**Minimum distance between flakes, d_{min} (μm)**	2.54	1.54
**The number of flakes per bacteria, Nb (10^6 bacteria per 1 mL)**	17700	118000

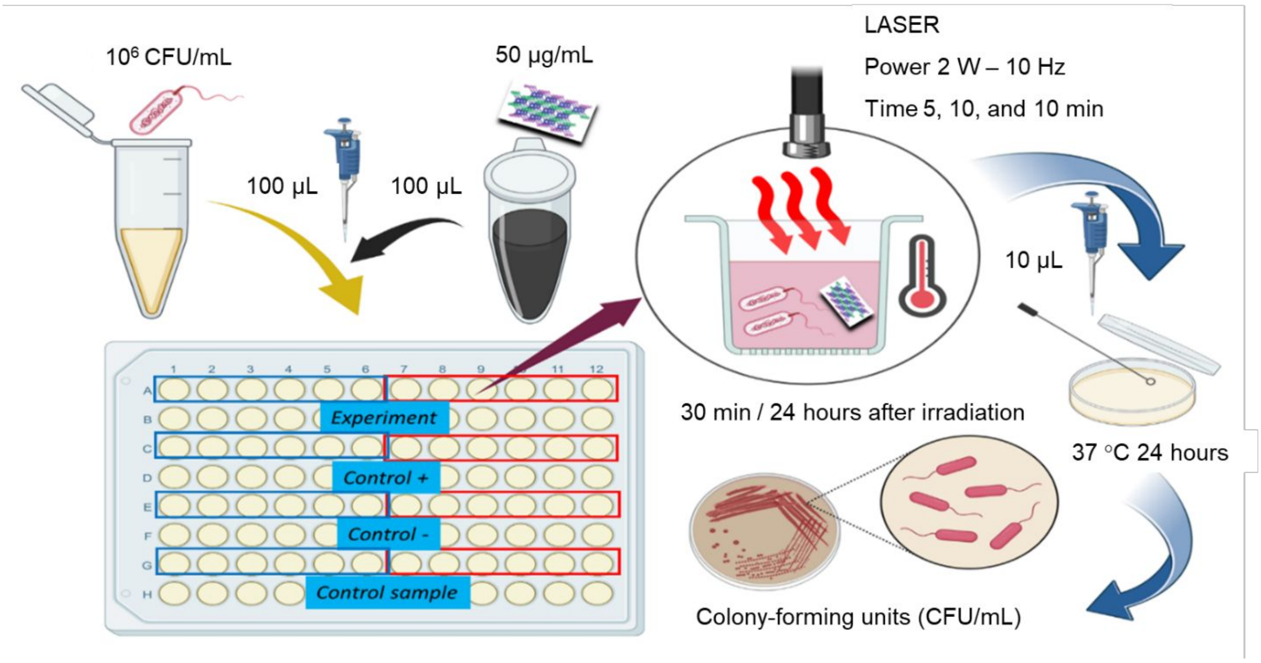

Figure S4. Schematic representation of MXene-assisted photothermal ablation.

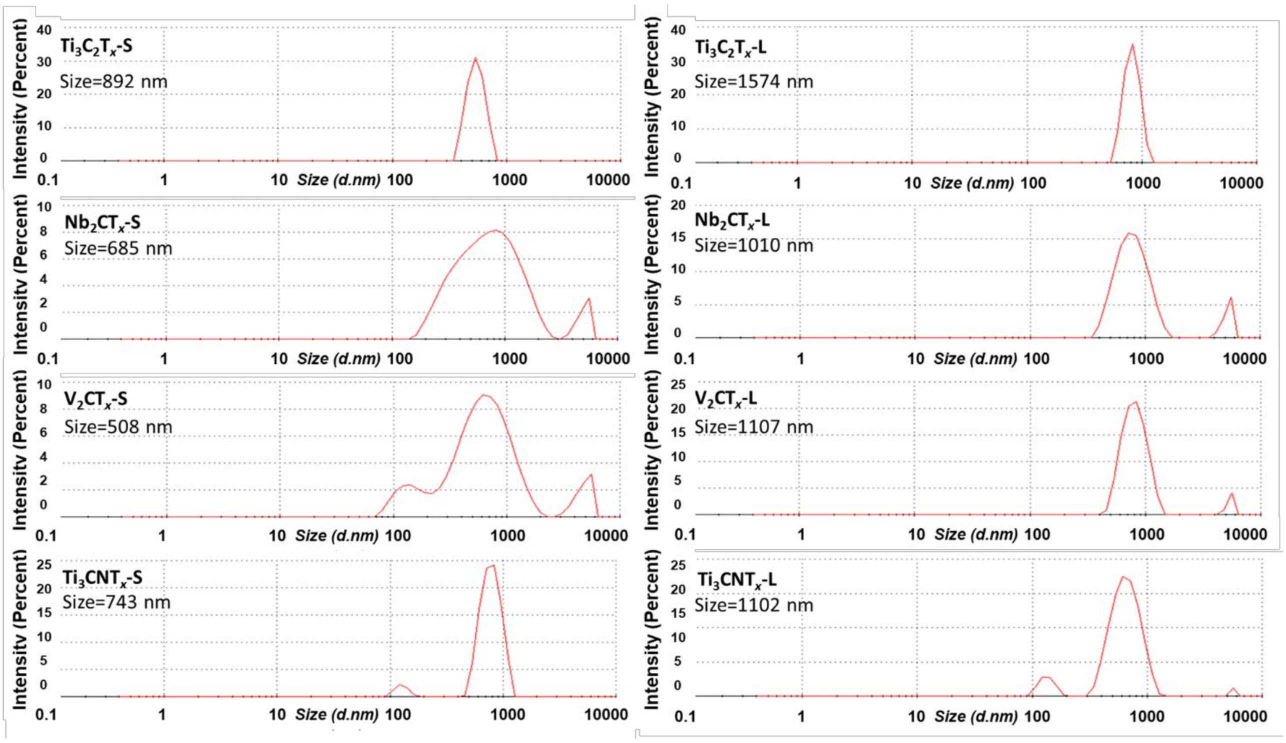

Figure S5. Particle size distributions of $Ti_3C_2T_x$, Nb_2CT_x, V_2CT_x, and Ti_3CNT_x MXenes based on light scattering measurements.

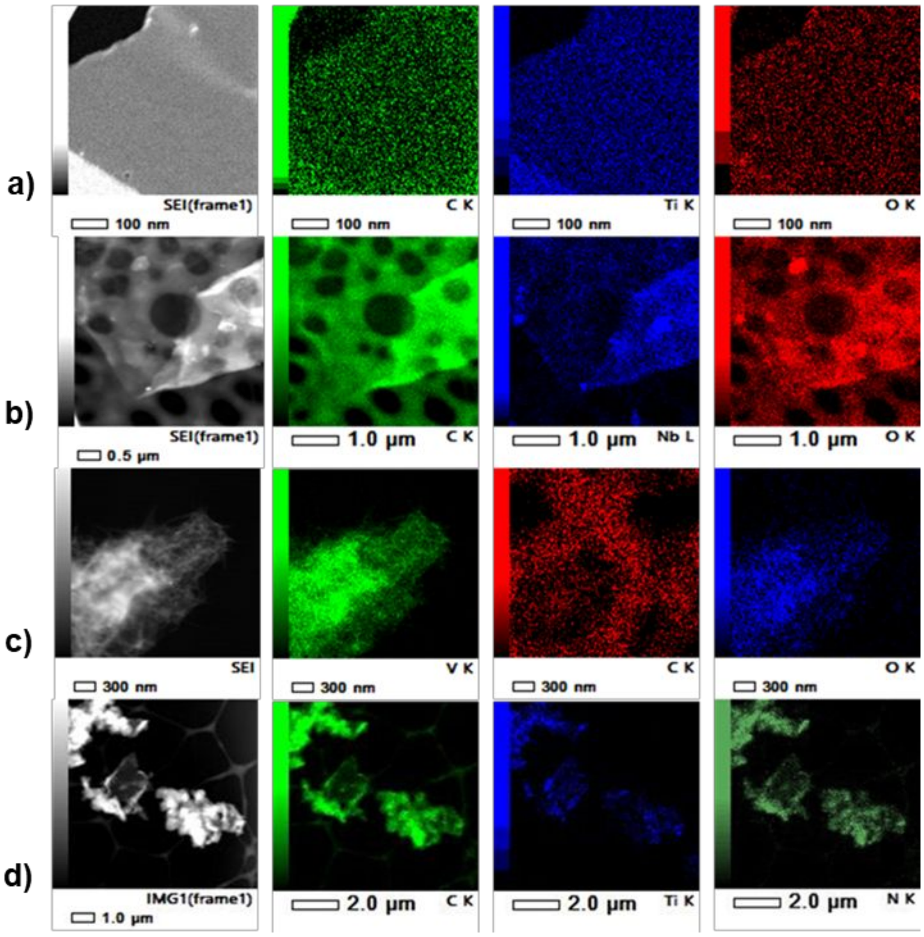

Figure S6. TEM images and EDS maps for a) $Ti_3C_2T_x$, b) Nb_2CT_x, c) V_2CT_x and d) Ti_3CNT_x.

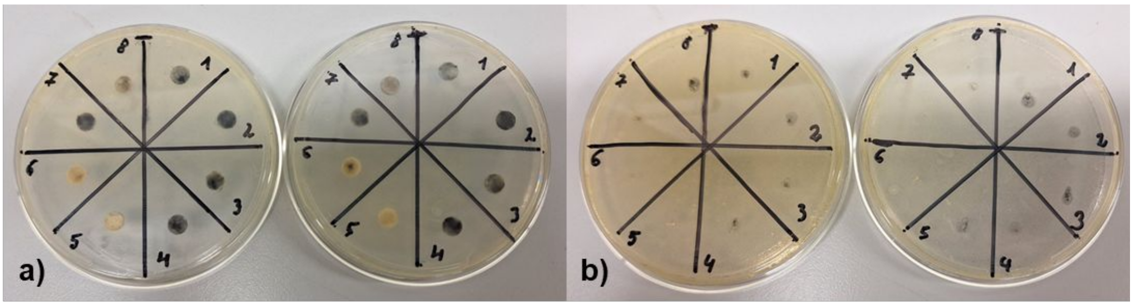

Figure S7. Disk diffusion method, 20 µL per disk in concentration of 2000 µg/mL (a) and drop diffusion method, 3 µL drop in concentration of 2000 µg/mL (b) for evaluation of MXene activity on *S. aureus* and *E. coli* (1 - $Ti_3C_2T_x$-S, 2 - $Ti_3C_2T_x$-L, 3 - Nb_2CT_x-S, 4 - Nb_2CT_x-L, 5 - V_2CT_x-S, 6- V_2CT_x-L, 7 - Ti_3CNT_x-S, 8 - Ti_3CNT_x-L).

Sample	Size	MIC, *μg/mL*		MBC, *μg/mL*	
		S. aureus	E. coli	S. aureus	E. coli
$Ti_3C_2T_x$	**S**	>	>	>	>
	**L**	>	1000	>	2000
Nb_2CT_x	**S**	1000	1000	2000	1000
	**L**	>	>	>	>
V_2CT_x	**S**	1000	1000	2000	1000
	**L**	2000	2000	>	2000
Ti_3CNT_x	**S**	>	1000	>	2000
	**L**	>	>	>	>

a)

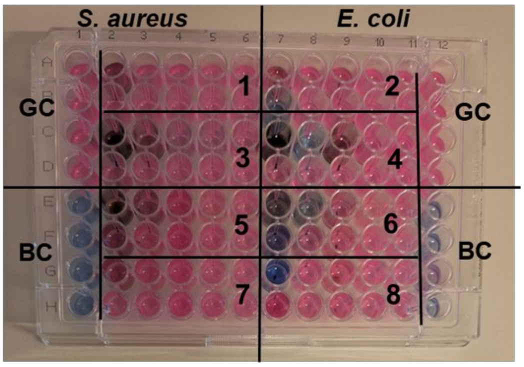

b)

Figure S8. Minimum Inhibitory Concentration (MIC) and Minimum Bactericidal Concentration (MBC) of MXene against *S. aureus* and *E. coli*; (b) change in the color of resazurin (1 - $Ti_3C_2T_x$-S, 2 - $Ti_3C_2T_x$-L, 3 - Nb_2CT_x-S, 4 - Nb_2CT_x-L, 5 - V_2CT_x-S, 6- V_2CT_x-L, 7 - Ti_3CNT_x-S, 8 - Ti_3CNT_x-L). GC – Growth control, BC – Broth control.

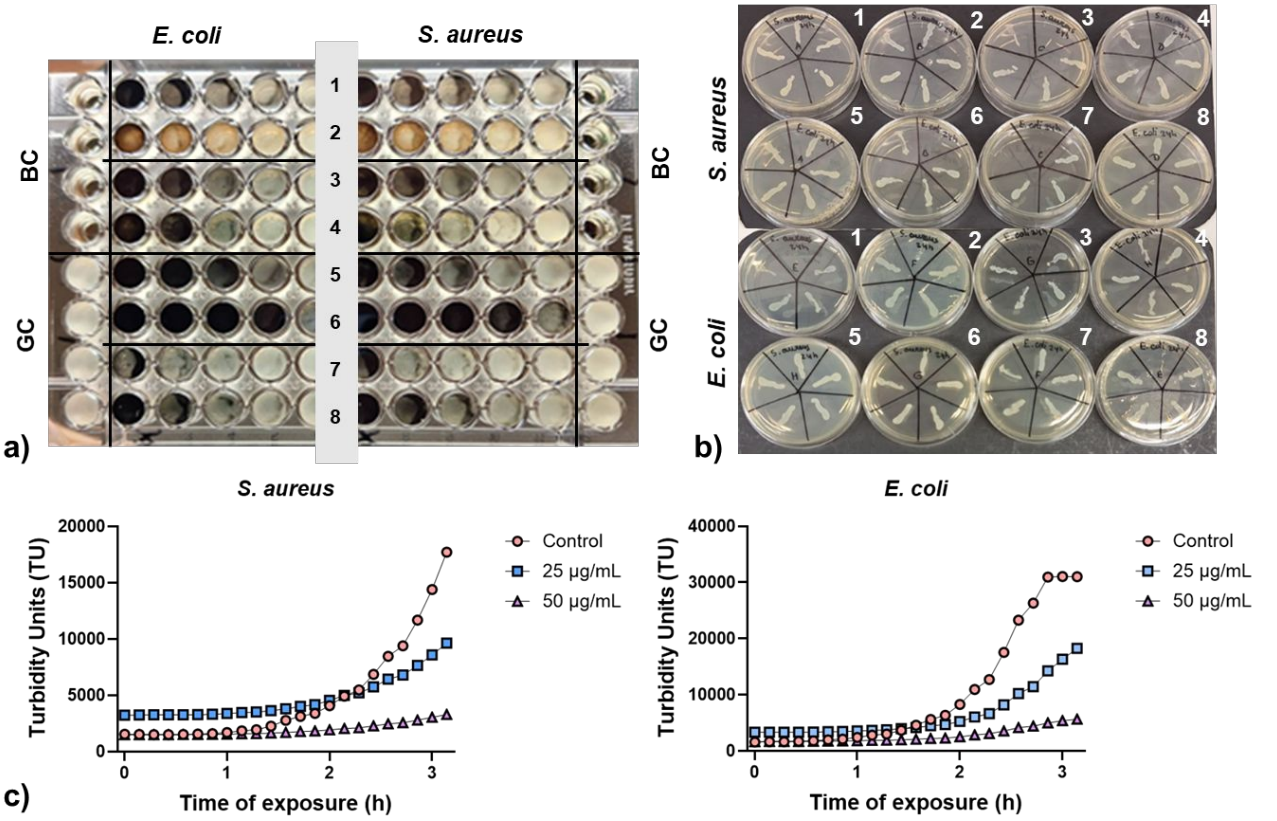

Figure S9. MIC and MBC assessment of various MXenes: (a) Bottom view of the 96-well plate after 24 hours of co-cultivation used to determine the minimum inhibitory concentration (MIC) of the tested MXenes. (b) Representative image of the replica plating performed for the minimum bactericidal concentration (MBC) assessment after 24 hours of co-cultivation. Petri dishes are divided into sectors, each corresponding to a specific MXene concentration, aligned with the respective wells of the 96-well plate. (c) Comparative Growth of *S. aureus* and *E. coli* in the presence of $Ti_3C_2T_x$ MXene over 24 hours of co-incubation assessed by HB&L Nephelometry (data for 200 μg/mL MXene are not presented, as the turbidity of the sample was beyond the instrument's detection capacity). Sample labeling: 1 - $Ti_3C_2T_x$-S, 2 - $Ti_3C_2T_x$-L, 3 - Nb_2CT_x-S, 4 - Nb_2CT_x-L, 5 - V_2CT_x-S, 6- V_2CT_x-L, 7 - Ti_3CNT_x-S, 8 - Ti_3CNT_x-L.

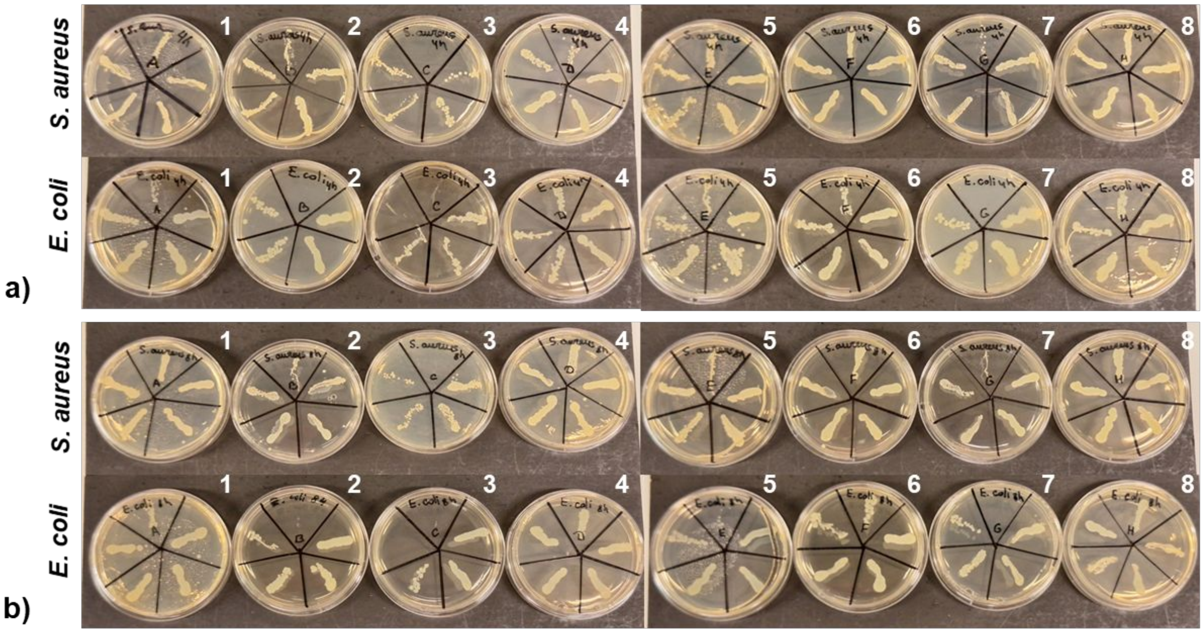

Figure S10. Time-dependent antibacterial activity of MXenes at varying concentrations (2000, 1000, 500, 250, and 125 μg/mL): Visual representation of colony growth of S. aureus and E. coli in Petri dishes divided into sectors, each corresponding to a specific MXene concentration aligned with the respective wells of a 96-well plate. Sector labels (A, B, C, D, etc.) denote the corresponding rows in the plate. Bacterial growth was assessed after 4 h (a) and 8 h (b) of incubation. Sample labeling: 1 - $Ti_3C_2T_x$-S, 2 - $Ti_3C_2T_x$-L, 3 - Nb_2CT_x-S, 4 - Nb_2CT_x-L, 5 - V_2CT_x-S, 6- V_2CT_x-L, 7 - Ti_3CNT_x-S, 8 - Ti_3CNT_x-L.

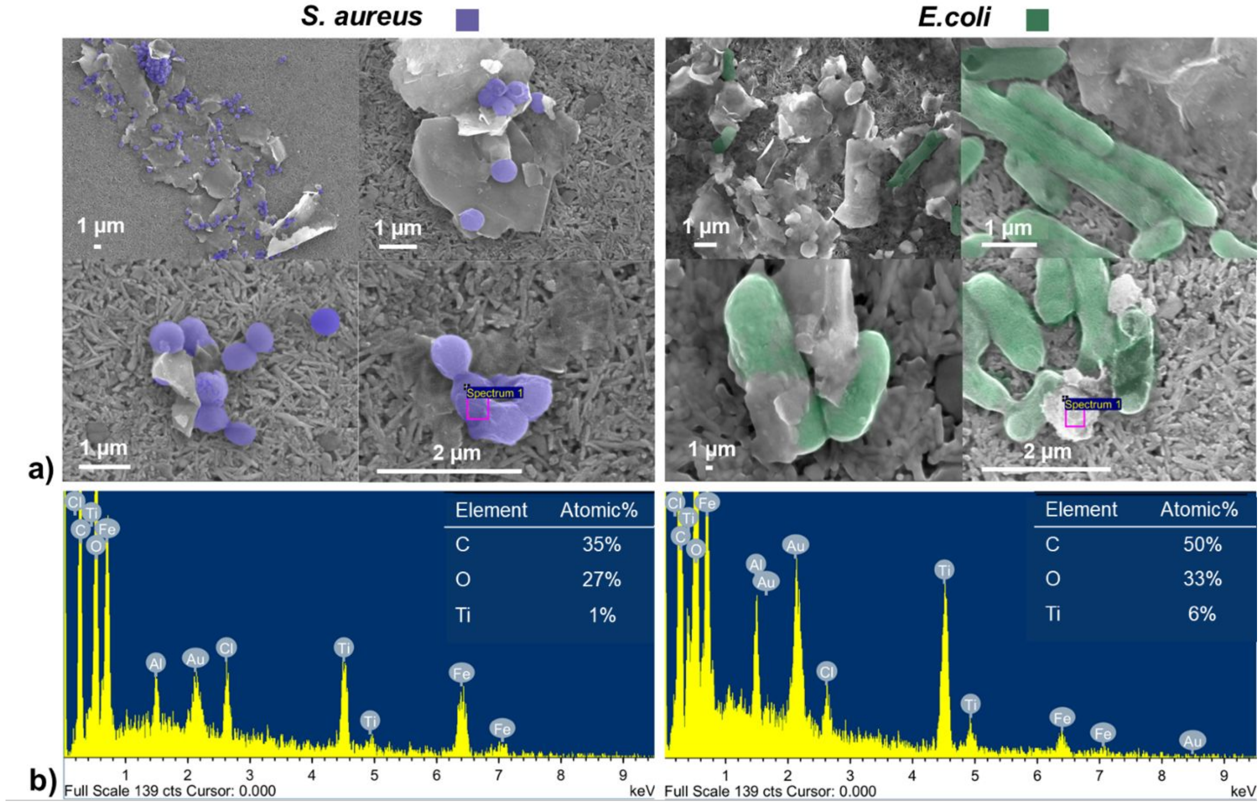

Figure S11. (a) SEM images of the *S. aureus* and *E. coli* treated with 2000 μg/mL of $Ti_3C_2T_x$ MXenes, at low and high magnification, respectively. The squares indicate the areas of EDX analysis (bacteria were artificially colored for easy recognition) (b) for determining the presence of element distribution (at. %), where Au originates from sputter coating and Fe from the electrically conductive substrate.

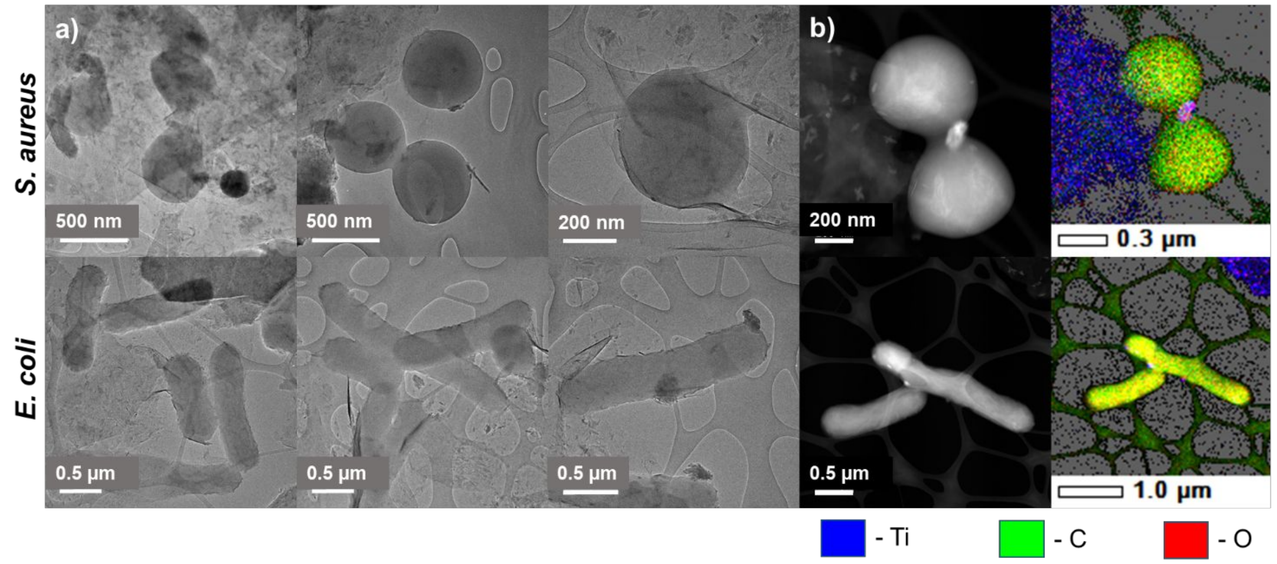

Figure S12. (a) TEM images of the *S. aureus* (top panel) and *E. coli* (bottom panel) treated with 2000 μg/mL of $Ti_3C_2T_x$ MXene with (b) EDX maps of elemental distributions of Ti, C and O.

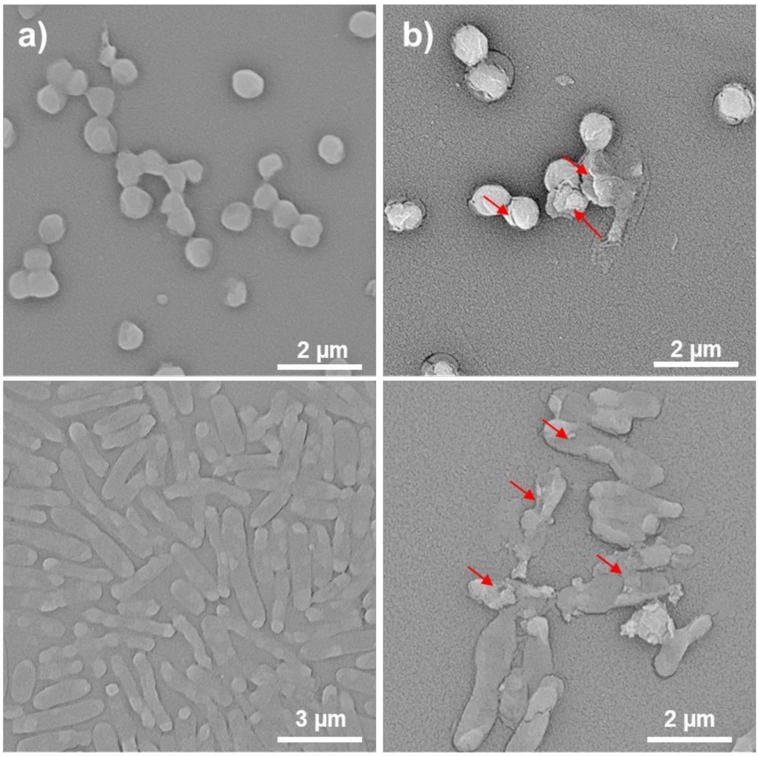

Figure S13. SEM images of the *S. aureus* (top panel) and *E. coli* (bottom panel) after $Ti_3C_2T_x$ MXene-based photothermal therapy (a) - control samples; (b) strains treated with $Ti_3C_2T_x$ MXenes. Red arrows indicate morphological changes in bacterial cells (surface cracks, membrane indentations, cytoplasmic leakage).

**R1**	8msec 10j/cm2 1.0Hz (10+10+10s)
**R2**	8msec 10j/cm2 1.0Hz (20s)
**R3**	8msec 10j/cm2 1.0Hz (15s)
**R4**	8msec 10j/cm2 1.0Hz (5+5+5s)
**R5**	8msec 10j/cm2 1.0Hz (5s)
**R6**	8msec 10j/cm2 1.0Hz (10+10s)
**R7**	8msec 10j/cm2 1.0Hz (5+5s)
**R8**	8msec 10j/cm2 1.0Hz (7s)
**R9**	8msec 10j/cm2 1.0Hz (8s)
**R10**	8msec 10j/cm2 1.0Hz(10s)
**R11**	7msec 12j/cm2 1.0Hz (5s)
**R12**	7msec 12j/cm2 1.0Hz (10s)
**R13**	7msec 12j/cm2 1.0Hz (15s)
**R14**	7msec 18j/cm2 1.0Hz (5s)
**R15**	7msec 10j/cm2 1.0Hz (20s)
**R16**	7msec 10j/cm2 1.0Hz (25s)
**R17**	7msec 5,9j/cm2 1.0Hz (30s)
**R18**	7msec 5,9j/cm2 1.0Hz (5s)
**R19**	7msec 5,9j/cm2 1.0Hz (10s)
**R20**	7msec 5,9j/cm2 1.0Hz (15s)
**R21**	7msec 5,9j/cm2 1.0Hz (20s)
**R22**	7msec 5,9j/cm2 1.0Hz (25s)
**R23**	7msec 5,9j/cm2 1.0Hz (10+10+10s)
**R24**	7msec 5,9j/cm2 1.0Hz (10+10s)

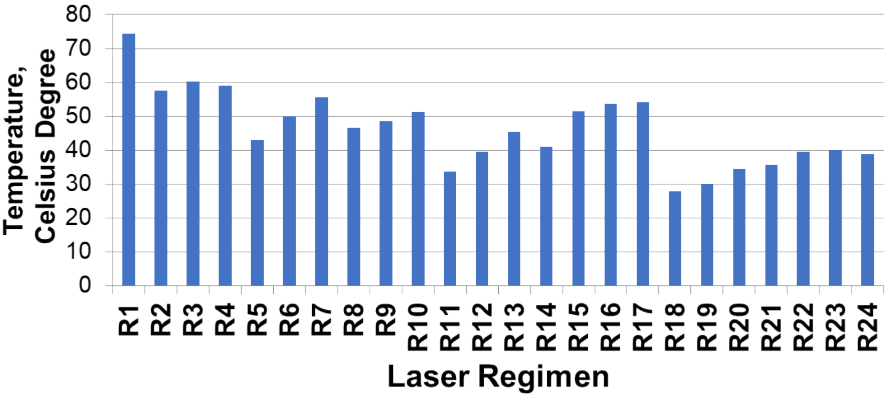

Figure S14. Laser regimens with the representative temperature growth after the NIR irradiation.

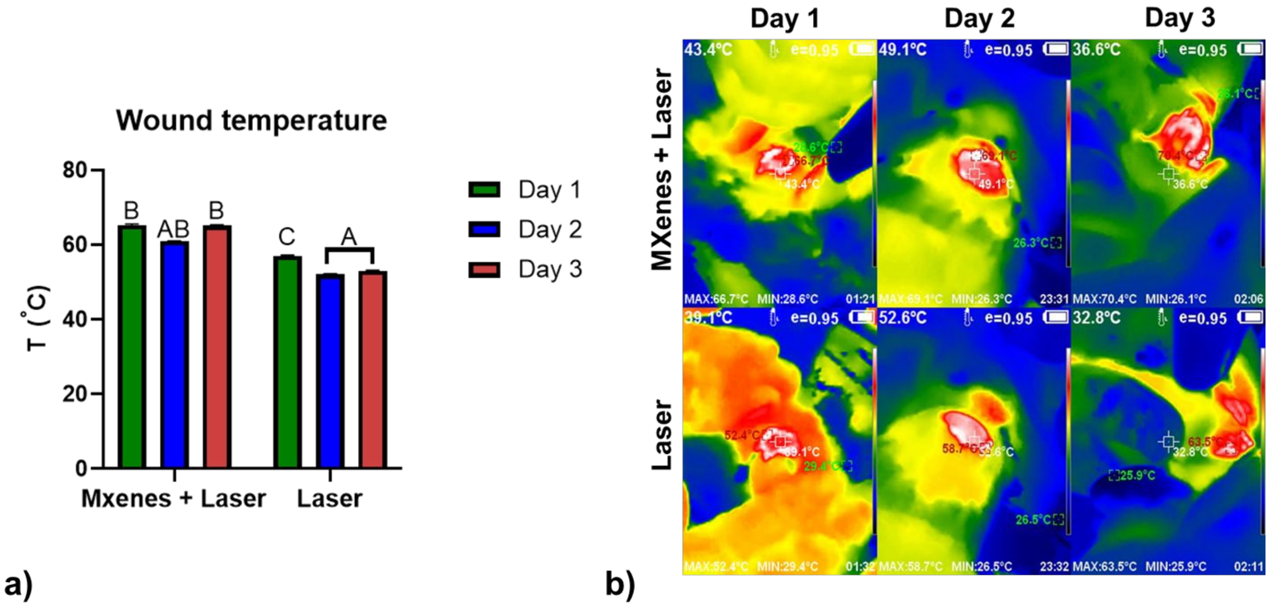

Figure S15. Dynamics of wound temperature changes (a) with the thermal images of a wound (b) under the influence of laser irradiation.
